# Fundus-controlled perimetry (microperimetry): Application as outcome measure in clinical trials

**DOI:** 10.1016/j.preteyeres.2020.100907

**Published:** 2020-10-03

**Authors:** Maximilian Pfau, Jasleen Kaur Jolly, Zhichao Wu, Jonathan Denniss, Eleonora M. Lad, Robyn H. Guymer, Monika Fleckenstein, Frank G. Holz, Steffen Schmitz-Valckenberg

**Affiliations:** a Department of Ophthalmology, University of Bonn, Bonn, Germany; b Department of Biomedical Data Science, Stanford University, Stanford, USA; c Nuffield Department of Clinical Neurosciences, University of Oxford, Oxford, UK; d Centre for Eye Research Australia, Royal Victorian Eye and Ear Hospital, East Melbourne, Victoria, Australia; e Ophthalmology, Department of Surgery, The University of Melbourne, Melbourne, Victoria, Australia; f University of Bradford, UK; g Department of Ophthalmology, Duke University Medical Center, Durham, NC, USA; h John A. Moran Eye Center, University of Utah, USA

**Keywords:** Retina, Microperimetry, FCP, Age-related macular degeneration, Inherited retinal diseases, Inherited retinal dystrophy, Functional outcome measures

## Abstract

Fundus-controlled perimetry (FCP, also called ‘microperimetry’) allows for spatially-resolved mapping of visual sensitivity and measurement of fixation stability, both in clinical practice as well as research. The accurate spatial characterization of visual function enabled by FCP can provide insightful information about disease severity and progression not reflected by best-corrected visual acuity in a large range of disorders. This is especially important for monitoring of retinal diseases that initially spare the central retina in earlier disease stages. Improved intra- and inter-session retest-variability through fundus-tracking and precise point-wise follow-up examinations even in patients with unstable fixation represent key advantages of these technique. The design of disease-specific test patterns and protocols reduces the burden of extensive and time-consuming FCP testing, permitting a more meaningful and focused application. Recent developments also allow for photoreceptor-specific testing through implementation of dark-adapted chromatic and photopic testing. A detailed understanding of the variety of available devices and test settings is a key prerequisite for the design and optimization of FCP protocols in future natural history studies and clinical trials. Accordingly, this review describes the theoretical and technical background of FCP, its prior application in clinical and research settings, data that qualify the application of FCP as an outcome measure in clinical trials as well as ongoing and future developments.

## 1. Introduction

Scientific and engineering developments in the past five decades provide us today with fundus-controlled perimetry (FCP, also called ‘microperimetry’) devices, allowing for feasible measurement of visual sensitivity and fixation stability in clinical practice and research. Previous manuscripts have described the diversity of applications to better screen for, diagnose and follow retinal diseases. However, a detailed explanation of the psychophysical and psychometric foundations underlying FCP, systematic classification of previous clinical findings and identification of areas that warrant further consideration are lacking in the current literature. In the present work, we review and describe FCP technology (1. History and devices, 2. Theoretical and technical background), its use in a clinical and research setting (3. Fixation and its examination, 4. Previous applications in studies), mode of application (5. Best practices) and next steps in its development and future use (6. Future directions).

### 1.1. Terminology

Whilst FCP (or fundus, fundus-driven perimetry) is commonly referred to as “microperimetry”, it must be noted that the stimulus sizes typically used are identical to stimuli applied in standard automated perimetry (Rohrschneider et al., 2008). Here, we use the term fundus-controlled perimetry (“FCP”) as originally suggested (Kani and Ogita, 1979).

### 1.2. History

#### 1.2.1. First description of the concept

In 1851, Hermann von Helmholtz not only revolutionized the field of ophthalmology with the invention of the ophthalmoscope, but he also provided the first description of FCP in the very same publication (Helmholtz, 1851). Using the ophthalmoscope both as viewing system to track the fundus and its inherent light as stimulus, von Helmholtz demonstrated that the optic nerve head does indeed correspond with a scotoma (Helmholtz, 1851). Based on this seminal observation, von Helmholtz concluded that visual phototransduction must inevitably occur prior to the optic nerve (Helmholtz, 1851).

#### 1.2.2. History of visual field testing

Subsequent developments of clinical visual field testing have laid the foundation for today’s standard automated perimetry as well as FCP. The clinical relevance of peripheral and central visual field testing was underscored by Albrecht von Graefe in 1856 in a hallmark publication that described in detail many clinical phenotypes of visual field loss including glaucomatous visual field loss, concentric visual field defects in retinitis pigmentosa, as well as paracentral ring scotomas in macular diseases and their implications for activities of daily living (v. Graefe, 1856). Soon after, Aubert and Förster laid the foundation for quantitative visual field testing using initially a tangent screen (Aubert and Foerster, 1857), and later by introducing the Förster (arc) perimeter to ensure a constant visual angle of the stimulus (Förster, 1869). The clinical importance of the central visual field and relative scotomas was highlighted in 1889 by Jannik Bjerrum. In his landmark paper on the diagnosis of glaucoma, he used a tangent screen and small ivory test-objects of variable size (Bjerrum, 1889). Ferree and Rand identified key determinates of differential light sensitivity and measurement reproducibility (Ferree and Rand, 1922), that provided the basis for the development of the Goldmann bowl perimeter (Goldmann, 1945, 1946). Standards set by this device including stimulus sizes and background illumination level for photopic testing were adapted for today’s commercially-available FCP devices. Earlier, Louise Sloan introduced the method of static perimetry as part of a comprehensive four-part discourse on clinical perimetry (Sloan, 1940), which led to the development of the Tübinger perimeter of Harms and Aulhorn designed for static perimetry in 1955 (Aulhorn and Harms, 2015). Further key developments were the automation of static perimetry (Heijl and Krakau, 1975; Spahr and Fankhauser, 1974), the introduction of visual field indices for the mathematical description of test results (Flammer et al., 1985; Heijl et al., 1987a), and the adoption of reliability measures including quantification of fixation losses (Heijl and Krakau, 1975) as well as false-positive or negative catch trials (Heijl et al., 1987b).

#### 1.2.3. History of FCP

Similar to von Helmholtz’s experiment, clinicians started documenting retinal sensitivity while examining the fundus (Meyers, 1959; Trantas, 1955; Watzke and Allen, 1969). In 1977, two devices were presented at the *“Second International Visual Field Symposium”* enabling FCP, however without automated fundus-tracking (Kani, 1977; Tagami and Isayama, 1977). A subsequent key development has been the invention of the scanning laser ophthalmoscope (Webb and Hughes, 1981), which facilitates high contrast imaging of the retina and was soon shown to be useful for FCP (Timberlake et al., 1982). Sunness and coworkers modified a fundus-camera to perform dark-adapted FCP (Sunness et al., 1988, 1987). Further key innovations toward clinically applicable FCP were the development of software for static threshold testing across multiple test-points (Rohrschneider et al., 1995b), and the introduction of fundus-tracking to enable point-by-point correlation and minimize the intra- and inter-session retest-variability through accurate stimulus placement (Sunness et al., 1995).

### 1.3. Commercially available instruments

The first commercially available device enabling FCP was the scanning laser ophthalmoscope 101 (SLO 101, Rodenstock Instrumente GmbH, Ottobrunn-Riemerling, Germany) presented at the *“Ninth International Visual Field Symposium”* (Plesch and Klingbeil, 1989; Stürmer et al., 1991). It projects a Helium–Neon laser beam (632.8 nm) and infrared diode laser (780 nm) concurrently on the fundus with a field size of 33 ° × 21 °. The HeNe-laser served as light source for the background illumination and stimulus presentation. While the SLO 101 permitted the presentation of variegated stimulus patterns in principle, it only allowed for presentation of red stimuli and the original Rodenstock software was insufficient for clinical use. This led to the development of multiple custom software solutions (Rohrschneider et al., 1995b).

The Micro Perimeter 1 (MP-1, NIDEK Technologies Srl, Padova, Italy) was the first commercially available device dedicated primarily to FCP. An infrared fundus camera (45° field of view) serves for imaging, while stimuli and the background are projected by a liquid crystal display (LCD) for mesopic testing (Midena et al., 2004; Springer et al., 2005). Moreover, its sophisticated software encompasses eye-tracking at a frequency of 25 Hz, allows for follow-up examinations, and offers the ability to (easily) customize the test-grid as well as test-parameters such as the stimulus size, duration and color. In 2011, Crossland and collaborators described a modification allowing for scotopic testing with the MP-1 (Crossland et al., 2011b), which led to the commercialization of the MP-1S featuring the addition of a neutral density filter and short-pass filter to the optical path. The device also allows for non-mydriatic color fundus photography. Disadvantages of the MP-1 device were the limited dynamic range of the liquid crystal display, which results in a marked ceiling effect in normal subjects or patients with mild functional loss (Bowl et al., 2013), and long testing duration in patients with poor fixation or large scotomas. For scotopic testing with the MP-1S, a “filter-selection-test” is necessary to select an appropriate neutral density filter in consideration of the limited dynamic range (Steinberg et al., 2017). Further, the threshold measurements along the vertical meridian were reported to be incompatible with the established hill-of-vision (Springer et al., 2005). Moreover, the IR-camera image quality is suboptimal. The device (now replaced by the MP3 device, cf. below) was nevertheless a major step forward and has been used in various large-scale multicenter trials (Holz et al., 2018).

The OCT/SLO microperimeter (Optos plc, Dunfermline, U.K. [previously OPKO Instrumentation, Miami, FL]) represent another FCP device that is no longer commercially available. The unit performs optical coherence tomography, scanning laser ophthalmoscopy and FCP. The system uses an organic light-emitting diode (OLED) screen to present stimuli on a photopic background (10 cd/m^2^). While this instrument facilitates structure function correlation, the limited dynamic range of 20 dB impacts its clinical applicability (Liu et al., 2014).

The Macular Integrity Assessment device (MAIA, CenterVue S.p.A., Padova, Italy) addressed technical limitation in tracking, image quality and dynamic range of earlier devices. Its SLO system provides a high image quality for fundus-tracking (and for subsequent structure function correlation). For stimulus presentation, the MAIA uses a light emitting diode (LED) and provides a dynamic range of 36 dB for mesopic testing (background 1.27 cd/m^2^). No ceiling effect is present for this instrument and the sensitivity profiles in healthy subjects match the established photoreceptor distribution (Pfau et al., 2017a). The S-MAIA (Scotopic Macular Integrity Assessment, CenterVue) device enabling dark-adapted two-color testing with an additional cyan and red LED has been developed subsequently (Pfau et al., 2017a). The dynamic range for dark-adapted testing has been extended from initially 20 dB–36 dB for the final version of the S-MAIA device (Pfau et al., 2020c). While the S-MAIA provides both scotopic and mesopic testing, none of the MAIA devices allow for photopic testing.

Similar to the MP-1, the MP-3 (NIDEK CO., Ltd., Gamagori, Japan) uses, an infrared fundus camera for tracking and an LCD projector for stimulus presentation. This projector allows for both mesopic (1.27 cd/m^2^) and photopic (10 cd/m^2^) testing and features a dynamic range of 34 dB ensuring the absence of a ceiling effect. A scotopic version of the device is now also available (NIDEK MP-3 Type S). The MP-3 also allows for high-resolution, non-mydriatic color funds photography and features the highest tracking speed of the commercially-available devices (30 Hz).

The Compass (CenterVue) also allows for photopic (background of 10 cd/m^2^) FCP with a testing field of up to 60°. The device is based on a SLO system for fundus-tracking and uses an LED to project the stimuli.

In addition to commercially available devices, custom built devices have been designed by various research groups to answer specific research questions. This includes adaptive-optic (AO) FCP devices allowing for single-cell psychophysics (Harmening et al., 2014).

## 2. Theoretical and technical background

### 2.1. Psychophysics and fundamentals of perimetry

The theoretical framework underlying FCP is largely based on standard automated perimetry and fundamental psychophysical principles. However, some factors such as the fundus-tracking and the Maxwellian view setup are unique to FCP. This section provides the fundamental principles that also govern the provided best-practice recommendation below (cf. 5. Best practices).

#### 2.1.1 Wb’s law, Fechner’s law and the decibel scale

2.1.1.

The decibel scale employed in FCP has its foundation in Weber’s law and Fechner’s law. The general relationship between the initial intensity of a given stimulus (e.g. luminance in the case of light) and the smallest detectable increment was formalized by Weber. According to Weber’s law, the just-noticeable difference (JND), the smallest change in stimulus (ΔL) that can be perceived, is proportional to the initial stimulus intensity (L).

$$\text{const.}=\frac{\Delta L}{L}$$

Based on the assumption that Weber’s law holds (i.e., the JND is a constant fraction of the stimulus), and that the JND is the basic unit of perceived magnitude (all JND steps are perceptually equal to each other), Fechner’s law stated that the subjective sensation is proportional to the logarithm of the stimulus intensity. For visual field testing, the logarithmic unit decibel (dB) has been adapted to express the threshold differential luminance (Δ*L* = *L*_*Threshold*_ – *L*_*Background*_) in relation to the instrument-specific maximum stimulus luminance (Lmax). It is defined according to the International Organization for Standardization (ISO 12866:1999) as:

$$\Delta S\left(in\;dB\right)=10\times {\text{log}}_{10}\frac{L_{\text{max}}}{\Delta L}$$

Therefore, 0 dB denotes the brightest stimulus a device can display. Each 1 dB increment corresponds to an attenuation of the stimulus luminance by a factor of 1.26, while 3 dB corresponds approximately to an attenuation factor of 2-fold and 10 dB corresponds to an attenuation factor of 10-fold. Due to the logarithmic scaling, decibel values between different devices (with the same background luminance) are theoretically convertible by simple addition or subtraction of a constant if the maximum stimulus luminance (i.e., luminance of 0 dB stimulus) differs (cf. Supplementary Table S1 and 2.4.3 Inter-device reliability).

#### 2.1.2. Differential luminance sensitivity or threshold luminance

The response probability in dependence of the stimulus intensity is a continuous, monotonous function. It may be described by the psychometric function relating stimulus intensity to response probability of sigmoid shape. Hereby, the differential luminance sensitivity (DLS, or threshold luminance) is defined as the stimulus intensity corresponding to a response probability of 50% (Schiefer et al., 2005).

#### 2.1.3 Static perimetry and threshold (staircase) strategies

2.1.3.

In static perimetry, the retinal sensitivity is probed at fixed test-points as defined by the perimetry test-pattern. Various threshold strategies may be used for static perimetry testing including strategies such as the 4–2 staircase strategy. These aim to identify the threshold at which a stimulus has a 50% probability of being detected. The accuracy and test duration of staircase strategies is dependent on the start point, step-sizes and number of reversals (Schiefer et al., 2005). A further group of adaptive strategies commonly encountered in non-fundus controlled static perimetry are Bayesian and maximum-likelihood procedures. Today, the COMPASS device is the only commercially available fundus-controlled device that offers such a threshold strategy (ZEST).

A special case is supra-threshold testing with a fixed stimulus intensity. While supra-threshold testing is not informative of the threshold, it may be applied to map scotomas in a very time-efficient manner (Wu et al., 2019a).

#### 2.1.4 Kinetic perimetry

2.1.4.

Kinetic perimetry is an alternative method of perimetry. Hereby, stimuli are moved along a trajectory (termed vector) from areas of non-seeing to seeing until a response is recorded. Compared to static perimetry, this approach is more time-efficient for the assessment of the peripheral visual field, which is irrelevant in the setting of FCP due to the limited testing and imaging field. A second potential advantage is the possibility to map scotomas with high spatial-resolution. The SLO 101 (with custom software) and the MP-1 devices allow for automated kinetic perimetry while imaging the fundus (Rohrschneider et al., 2008, 1995a). However, to our knowledge, kinetic perimetry has not been applied in the setting of FCP study since.

#### 2.1.5 temporal summation and stimulus duration

2.1.5.

Bloch’s law states that the visual threshold is proportional to the product of the stimulus luminance and duration and applies up to a critical time (approximately 100 ms). In commercially available FCP devices, the stimulus duration is typically 200 ms and therefore beyond the temporal integration time. This ensures that minor inaccuracies in the duration of stimulus presentation do not affect threshold measurements. Stimulus durations longer than 200 ms, which is a possible setting for the MP-1 device, are generally not advisable given that the initiation of saccades to an unexpected stimulus takes about 200 ms (Fuchs, 1967).

#### 2.1.6 Spatial summation and stimulus size

2.1.6.

Ricco’s law states that the ratio of the stimulus luminance and angular area of a stimulus required for detection are constant for small targets that are within the receptive field of a single retinal ganglion cell (e.g., the Goldmann I and II stimuli) (Khuu and Kalloniatis, 2015a). For stimuli larger than the critical area (Ricco’s area), Piper’s law applies stating that ratio of the stimulus luminance and square-root of the angular area required for detection are constant (Khuu and Kalloniatis, 2015a). In optic neuropathies such as glaucoma, stimuli scaled to the size of Ac may be optimal to detect drop-out by small numbers of ganglion cells (Anderson, 2006; Khuu and Kalloniatis, 2015b). Nevertheless, stimuli larger than the critical area are often applied clinically to monitor glaucoma in consideration of the dynamic range and repeatability (Khuu and Kalloniatis, 2015b; Wall et al., 2010). These aspects have not been studied in detail in the context of FCP and macular diseases to date.

In FCP, typically Goldmann III stimuli (diameter of 0.43°) are used across devices, which are larger than the critical area in the central retina of healthy observers under photopic conditions (Anderson, 2006). For scotopic testing with the MP-1S device, Goldmann V stimuli (diameter of 1.72°) have previously been applied (Steinberg et al., 2017), which are also larger than the critical area for scotopic conditions in healthy observers (Anderson, 2006; Redmond et al., 2010).

#### 2.1.7 Reliability indices

2.1.7.

Multiple indices have been proposed to predict the reliability of a given examination (cf. with 2.4 Retest-reliability below). In standard automated perimetry, the quantification of so-called “fixation losses” (Heijl–Krakau “fixation catch-trial errors”) is done by presenting stimuli to the blind spot (Heijl and Krakau, 1975). In FCP, this method has also been integrated – however, due to fundus-tracking, this measurement represents not a true measure of fixation loss (despite of the name), but rather a false-positive catch trial (assuming accurate selection of the optic disc by the operator, no head tilt bringing the supposed blind spot into view, and sufficiently accurate fundus-tracking). Further, these catch-trials are typically presented at a low frequency (approximately 1/min). The estimate of these false positive responses might be imprecise due to the low sampling rate (i.e., the measured rate of “fixation losses” is likely to deviate from the true false-positive response rate for any given patient).

Another measure of patient reliability adopted from standard automated perimetry is the rate of wrong-press events (Olsson et al., 1997). These are defined as response button press events outside of the temporal response window, which the MAIA device records. The last generation of devices (COMPASS and MP-3) also offer conventional false-negative and false-positive catch trials.

For FCP, multiple groups have proposed the use of cut-off values for false-positive responses to stimuli to the optic nerve head of 25% (Wu et al., 2016a). Yet, factors related to the disease severity (e.g., mean sensitivity) appear to have an even greater influence on the retest-reliability (von der Emde et al., 2019b). There is continued interest in using fixation parameters as well as consideration of the test duration for the assessment of reliability and this is still under investigation (Yohannan et al., 2017).

#### 2.1.8 Dynamic range, ceiling and floor effects

2.1.8.

A major limitation of the first commercially available FCP devices is the limited dynamic range that can lead to false conclusions in the interpretation of test results. For FCP application, it is essential to consider the physical dynamic range (range of the dimmest to the brightest presentable stimulus) of the device with regards to ceiling as well as floor effects. While ceiling effects impair the ability to detect mild loss in retinal sensitivity, residual retinal sensitivity might not be detected because of floor effects. Of note, the physiological range of retinal sensitivity is higher for rod as compared to cone function. Finally, it is important to consider the limited dynamic range in relation to the test-retest variability. For the MP-1 device, the physical dynamic range of 20 dB (using Goldmann III stimuli) was indeed shown to be insufficient to resolve normal retinal sensitivity and therefore also mild functional loss (Bowl et al., 2013). In contrast, no ceiling effect is evident for the MAIA, the COMPASS and the MP-3 device, highlighting the comparative advantage of these devices for the detection of mild functional loss (Pfau et al., 2017a).

### 2.2. Unique characteristics of FCP

#### 2.2.1. Fundus-tracking

**Fig. 1.**
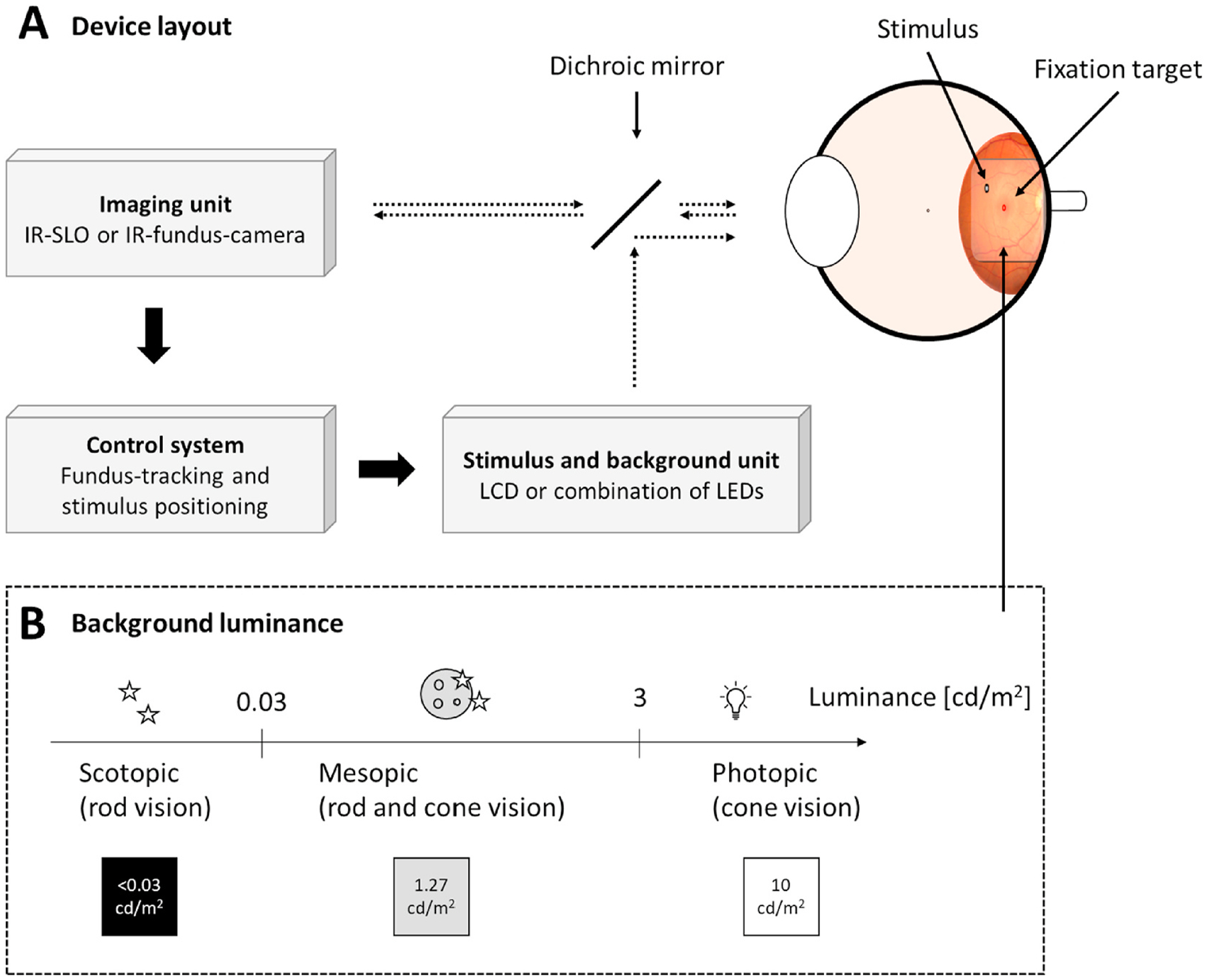
FCP devices.

The key feature of FCP is the ability to present stimuli at specific retinal locations enabled by continuous visualization of the retina throughout and between examinations (Fig. 1). This allows for (i.) testing of retinal sensitivity in patients without stable fixation, (ii.) repeated testing at the same location to improve the intra- and intersession reliability, which enhances longitudinal comparability of results, as well as (iii.) precise structure function correlation. Precise data on the *in vivo* tracking performance (especially of the devices in comparison to each other) is unfortunately not available. While manufacturers typically report the frequency of eye tracking, it is unclear how this translates to accuracy in stimulus presentation. For the MP-1 device, it was shown in an *ex vivo* setting that the time difference between movement of a tracking feature (simulating a saccade) and the corresponding onset of stimulus movement was 83 ± 12 ms (Cideciyan et al., 2016). In conjunction with eye movement data, the authors estimated that such a delay would result in an average absolute displacement error of <0.25° in patients lacking foveal fixation (end-stage retina-wide photoreceptor degeneration) (Cideciyan et al., 2016).

Overall, the retest-reliability appears to be more uniform (i.e., less heteroscedastic) compared to standard automated perimetry (SAP) (Pfau et al., 2017b). However, studies comparing FCP with and without activated fundus-tracking are lacking. Further, heteroscedasticity has been reported in patients with glaucoma (Wu et al., 2016b), Stargardt disease (Pfau et al., 2020b) as well as age-related macular degeneration (Barboni et al., 2018). Further, lower retest-reliability has been reported at the boundary of the optic nerve head as a model for deep scotomas (Wu et al., 2015b) and within areas of high local gradients of sensitivity (e.g. the location between a region of normal and markedly reduced sensitivities) (Wu et al., 2016b), with high spatial density testing. Whilst a direct comparison with non-fundus controlled perimetry has not been performed, the measurement variability for regions with severe loss of function are likely to be much lower with FCP.

#### 2.2.2. Maxwellian view versus natural viewing optical systems

FCP devices employ typically a Maxwellian view setup in contrast to standard automated perimetry (Midena and Pilotto, 2013), which means that the illumination source is made optically conjugate to the pupil of the eye (Burns and Webb, 1995). This leads to important implications with regard to the effect of pupil size on threshold determinations. In standard automated perimetry, the retinal illumination can vary depending on the pupil diameter and lenticular opacification. However, since the background and stimulus are equally affected by these variations and since Weber’s law holds under photopic conditions, the thresholds remain identical (Aulhorn et al., 1966). In contrast, for mesopic testing, minor reductions in light transmission may affect the measured thresholds (Klewin and Radius, 1986).

On the other hand, due the Maxwellian view setup of FCP, pupil size is not expected to affect threshold determinations even under mesopic conditions, since the pupil size required by the devices is designed to be smaller than the typical size of the human pupil. The absence of an effect of pupil dilatation has been experimentally confirmed for the MAIA device (Han et al., 2017). However, defocus and decentration due to clipping of the stimulus by the iris may affect the test results (cf. Fig. 3).

### 2.3. Photoreceptor specificity and dark-adapted testing

**Fig. 2.**
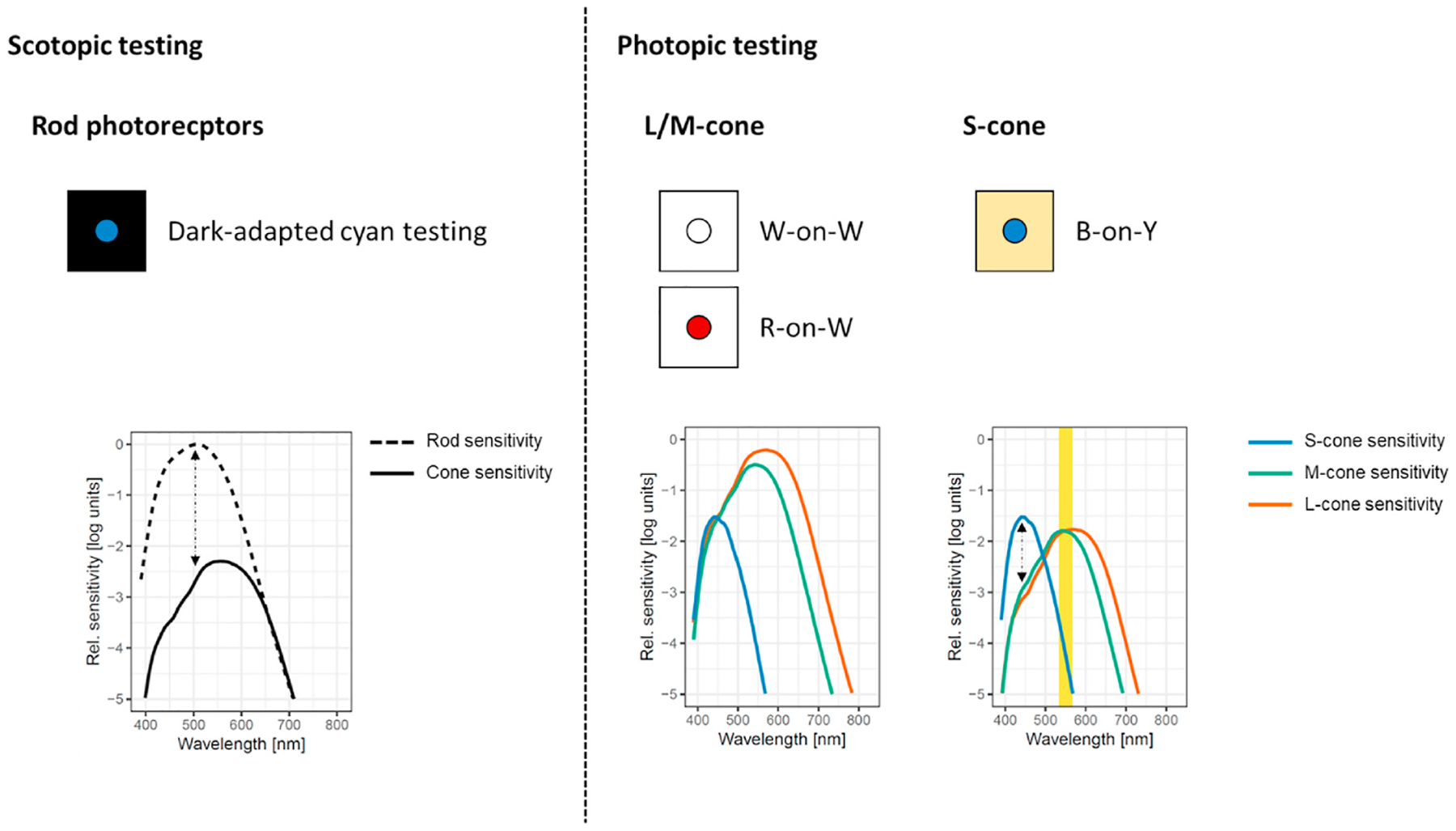
Selective perimetry strategies.

Isolation techniques for perimetry testing have been established, that take advantage of the differential spectral sensitivity of the S-, L/M-cone and rod photoreceptors (Fig. 2). Hereby, relative photoreceptor function isolation may be achieved through optimization of the stimulus and background. Importantly, photoreceptor function isolation is indispensable (i.) to measure functional loss or potential treatment effects in a specific and hypothesis-driven manner and (ii.) to avoid “redundancy” of target detection leading to insensitivity to potentially photoreceptor-specific lesions (Simunovic et al., 2016). However, mesopic white-on-white testing is the most common test condition used in FCP, which maximizes the redundancy of target detection (Simunovic et al., 2016). Instead of a psychophysical rationale, the choice of a mesopic background illumination of the MP-1 device appears to be linked to the highest practical illumination level with regard to the dynamic range of the built-in liquid crystal display.

Dark-adapted (DA, scotopic) testing presumably allows for probing relative rod compared to cone photoreceptor function using a single stimulus close to the peak of the scotopic sensitivity function. This is achieved with the MP-1S by incorporating a neutral density filter and a short-pass filter to the optical path (Crossland et al., 2011b). However, due to the limited dynamic range of the MP-1S, the neutral density filter must be adjusted depending on the degree of sensitivity loss of the patient (Steinberg et al., 2015). Further, this single stimulus technique does not allow documentation of the photoreceptor source, when rod sensitivities are reduced below normal DA cone sensitivities (McGuigan et al., 2016). The now available MP-3 Type S features a much greater dynamic range and therefore does not require a patient-specific selection of neutral density filters.

Dark-adapted two-color testing with cyan and red stimuli provides the ability to document the photoreceptor source of the measured thresholds based on the specific sensitivity difference (Ernst et al., 1983; Jacobson et al., 1986). The S-MAIA device implements this principle in the setting of FCP (Pfau et al., 2017a). However, the dynamic range of the current version of the device is limited effectively to 23 dB in consideration of the normal function (i.e., limits of perception), despite of the 36 dB physical dynamic range for DA testing allowing to present even dimmer stimuli (Pfau et al., 2020c).

Cone isolation can be achieved through photopic (10 cd/m^2^) white-on-white testing (cf. devices in Table 1, Fig. 2). Moreover, red-on-white perimetry and red-on-cyan perimetry or blue-on-yellow perimetry may be applied to isolate the L/M-cone or S-cone system, respectively (Luo et al., 2015; Simunovic et al., 2016, 2004). These principles, as of now, have only been rarely applied for fundus-controlled function testing (Cideciyan et al., 2016; Luo et al., 2015; Remky et al., 2001; Remky and Elsner, 2005).

### 2.4. Clinical endpoints and psychometric concepts

#### 2.4.1. Clinical outcome assessments

Clinical outcome assessment (COA) qualification is a regulatory requirement by the U.S. Food and Drug Administration (FDA). This is similar with other regulatory authorities around the world including the European Medicines Authority (EMA). The rules state that a COA must be a reliable and well-defined assessment of patients’ symptoms or function. Hereby, FCP testing would be classified as a performance outcome (PerfO). Measurement properties defined by psychometric test theory must be considered to establish the applicability of a PerfO in clinical trials. This includes reliability, validity and ability to detect change.

#### 2.4.2. Reliability

Multiple classes of reliability estimates are defined including (i.) intra- or inter-session test-retest reliability, (ii.) inter-method reliability, (iii.) inter-rater reliability and (iv.) internal consistency reliability. For FCP, intra-session test-retest reliability data is available for a variety of diseases, whereas inter-session test-retest reliability data is rare. However, for choroideremia, it has been shown that intra- and inter-session test-retest reliability are comparable for mesopic testing(Jolly et al., 2017). Similarly, disease-specific data on inter-method (i.e. inter-device) reliability is rare. The effect of the test administrator (inter-examiner variability) was shown to be insignificant in the setting of FCP (Weingessel et al., 2009). The concepts of inter-rater reliability and internal consistency reliability are not applicable for FCP.

#### 2.4.3. Inter-device reliability

Sensitivity measurements obtained with the same background should be convertible between the devices on the decibel scale through simple addition or subtraction of a constant (cf. 2.1.1 Wb’s law, Fechner’s law and the decibel scale).

However, this could not be confirmed empirically in an inter-device reliability comparing the MP-1 and MAIA device (Wong et al., 2016). Specifically, average differences (MAIA – MP-1, mesopic testing) were reported ranging from 7.3 dB (Wong et al., 2016), to 5.5 dB (Steinberg et al., 2017), which both exceed the theoretically expected average difference of 4 dB (Midena, 2014). In addition to the offset, Bland-Altman plots revealed that the difference between the MAIA and MP-1 device was less pronounced for test-points with low sensitivity and more pronounced with high sensitivity even when excluding test-points close to the floor and ceiling of the dynamic range (Wong et al., 2016). Comparison of retinal sensitivity measurements between the MAIA and MP-3 device (mesopic testing) revealed a 5.65 dB difference in healthy observers (Balasubramanian et al., 2017), despite of the similar specifications of these two devices (Supplementary Table S1). No specific data was reported for the inter-device reliability toward the lower end of the dynamic range (i.e., for patients with severe functional impairment).

Regarding photopic FCP testing with the COMPASS device, average differences (Humphrey field analyzer [HFA] - COMPASS) of 1 dB for healthy subjects and subjects with glaucoma (Rossetti et al., 2015), and of 1.85 dB for healthy subjects and of 1.46 dB for subjects with glaucoma (Montesano et al., 2019), were previously reported. This small difference is possibly explained by the difference of the applied threshold strategies in the respective studies (COMPASS: ZEST or 4–2 staircase, HFA: SITA) (Artes et al., 2002; Montesano et al., 2019; Rossetti et al., 2015).

For photopic testing with the MP-3 device, average differences (HFA – MP-3) of 4.8 dB (patients with glaucoma) and 4.9 dB (control subjects) were observed (Hirooka et al., 2016).

#### 2.4.4 Retest-reliability of FCP

2.4.4.

The Bland-Altman 95% coefficient of repeatability (COR or CR or Smallest Real Difference [SRD]) for point-wise results constitutes a meaningful measure of retest-reliability for FCP. The 95% COR, which is based on the within-subject retest-variance, describes the value below which the absolute differences between two measurements would lie with 0.95 probability (Martin Bland and Altman, 1986). Accordingly, point-wise changes exceeding this limit may therefore be considered as clinically significant (however, 5% of the test-points can be expected to show change beyond the 95% COR even in eyes with no change over time due to multiple testing). For mesopic testing using the MAIA device and a 4–2 staircase strategy, 95% COR of ±4.12 dB to ±4.52 dB were reported for diseases without deep scotomas such as intermediate age-related macular degeneration (Welker et al., 2018; Wu et al., 2013), and of ±5.79 dB to ±6.64 dB for diseases with deep scotomas such as geographic atrophy (Pfau et al., 2017b, 2020c; von der Emde et al., 2019b).

Similar point-wise 95% COR values of ±5.56 dB (with exclusion of floor and ceiling effects) and ±4.94 dB (without exclusion of test-points) were reported for the MP-1 device (Chen et al., 2009), as well as for the MP-3 device (±5.0 dB). However, values of 95% COR will vary with disease type and severity and should be investigated prior to any trials that aim to assess treatment effect.

Inter-session retest-reliability has been reported less frequently, however, it appears to be in a similar range as intra-session retest-reliability (Midena et al., 2010).

#### 2.4.5. Validity

There are multiple different forms of validity. Content validity describes how well a measure matches the construct it is meant to measure. Content validity of FCP could be established for some devices (e.g. MAIA) based on the sensitivity profile matching the human photoreceptor distribution (Pfau et al., 2017a). Criterion validity, which includes concurrent, discriminant, convergent and predictive validity, describes how well a measure relates to other characteristics and measures. The most common variant to establish concurrent validity in the setting of FCP is structure function correlation. Further, concurrent validity may be established by examining the association between FCP results and age or best-corrected visual acuity or change over time (Edwards et al., 2015; Hsu et al., 2019; Jolly et al., 2017). Examples for discriminant validity for FCP in early and intermediate AMD is the association of retinal sensitivity and the predominant drusen-subtype (Pfau et al., 2018a) and the disease stage (Cocce et al., 2018). The established association of the highest FCP sensitivity and full-field threshold (FST) in choroideremia would be an example of established convergent validity (Dimopoulos et al., 2018a).

#### 2.4.6. Ability to detect change (responsiveness, longitudinal construct validity)

Knowledge of the expected disease progression in terms of sensitivity loss over time (dB/year) constitutes a prerequisite for sample size considerations. Ability to detect change in the setting of FCP may be affected by the device in presence of a ceiling effect (inability to detect mild loss of function) or floor effect (inability to detect worsening of severe loss of function) as well as by the test-pattern in conjunction with the topographic manifestation of a disease. Responsiveness of FCP in patients undergoing anti-vascular endothelial growth factor therapy due to neovascular AMD is established (Bolz et al., 2010; Prager et al., 2008; Squirrell et al., 2010). However, there are relatively few natural-history studies examining the ability to detect change in atrophic retinal disease (Meleth et al., 2011; Etienne M. Schönbach et al., 2020b; Testa et al., 2014). Further, studies comparing the responsiveness of trend-based and event-based analysis are lacking.

#### 2.4.7. Clinically significant/meaningful change

While regulatory agencies including the U.S. FDA have expressed explicitly a preference for functional over anatomic endpoints (Csaky et al., 2017), no precise criteria defining a clinically meaningful visual field progression have been defined for FCP in retinal disease so far. In contrast, the U.S. FDA previously stated in the setting of SAP and glaucoma, that a statistically significant between-group mean difference of at least 7 dB (possibly also less) may be considered as clinically significant (Weinreb and Kaufman, 2009). For a given eye, progression of visual field loss may be defined by five or more visual field locations, which have significant changes from baseline beyond the 5% probability levels for the glaucoma-change-probability (GCP) analysis (De Moraes et al., 2017; Weinreb and Kaufman, 2009).

## 3. Examination of fixation

Fixation location and stability are recorded as a ‘byproduct’ of the fundus-tracking for stimulus presentations during FCP testing (Crossland and Rubin, 2014). The following sections will summarize previously applied types of fixation stability tests, quantitative metrics to describe fixation, disease-specific observations and how these measurements relate to other measures of visual function. Lastly, treatment of fixation instability through biofeedback training will be discussed.

### 3.1. Fixation testing

Fixation examination can be conducted in two fundamental manners. First, fixation characteristics may simply be recorded as part of the perimetry testing. Second, fixation characteristics may be acquired using a separate 10–30 s fixation test without perimetry testing (Crossland and Rubin, 2014; Longhin et al., 2013). These two types of tests have been previously referred to as ‘dynamic’ and ‘static’ examination of fixation (Etienne M. Schönbach et al., 2020a). Testing as part of the perimetry examination yields typically higher values for measures of fixation instability (Longhin et al., 2013; Etienne M. Schönbach et al., 2020a). Another important factor, which may influence the measured fixation stability, is the fixation target used. Specifically, using central targets yields significantly smaller values for measures of fixation instability as opposed to pericentral fixation targets such as the “4-point diamond” (Bellmann et al., 2004).

### 3.2. Fixation metrics

Fixation is summarized by a measure of central tendency (i.e., locus of fixation) and a measure of spread (i.e., fixation stability).

The locus of fixation, which is termed preferred retinal locus (PRL) for patients with extrafoveal fixation (Timberlake et al., 1987; von Noorden and Mackensen, 1962), is defined as the centroid of the individual fixation point measurements. However, some patients with central scotoma exhibit also multiple PRLs (Cummings et al., 1985; Duret et al., 1999; Lei and Schuchard, 1997), which may be chosen depending on the visual task (Crossland et al., 2011a). Crossland et al. proposed kernel density estimation of fixation point measurements to identify individual PRLs (M.D. Crossland et al., 2004). While this type of analysis may be performed using the raw data of the commercially available FCP devices, it is not integrated today. The MAIA provides two PRL locations, a PRL_i_ (i for initial), which describes the centroid of fixation for the initial 10 s of the test prior to perimetry testing (i.e., ‘static’ examination of fixation), and a PRL_f_ (f for final), which describes the centroid of all fixation point measurements during the exam (Morales et al., 2016). Differential PRL_i_ and PRL_f_ locations are prevalent in patients with unstable fixation (Morales et al., 2013).

To quantify and report fixation stability, two types of commonly applied metrics coexist as well as more recent metrics, which warrant further investigation. First, fixation stability may be quantified in relation to fixed circular regions centered to the PRL such as a circle with a radius of 1° or 2° (i.e., diameter of 2° and 4°, termed P1 and P2 for the MAIA device) (Morales et al., 2016). The so-called Fujii classification has been proposed, to categorize the fraction of fixation points within these regions as stable fixation (P1 includes greater than 75% fixation points), relatively unstable (P1 includes less than 75% and P2 includes more than 75% fixation points) and unstable (P2 includes less than 75% of fixations points) (Fujii et al., 2002). The early implementation of this classification in the MP-1 device and the simplicity contributed to a widespread adoption of the Fujii classification. However, it must be noted that this somewhat arbitrary discretization of a continuous phenomenon may lead to loss of relevant information.

A continuous measure of fixation stability is the bivariate contour ellipse area (BCEA) (Steinman, 1965). Typically, “global” BCEA values covering 63% and 95% of the fixation point measurements are provided (Morales et al., 2016; Steinman, 1965). Conventionally, these are log10 transformed for subsequent statistical analyses in consideration of the positive skew of BCEA values (Michael D. Crossland et al., 2004). As mentioned above for the PRL, a subset of patients exhibits multiple PRLs. The spread of fixation is inadequately quantified by a single BCEA fitted to the fixation patient measurements in such patients. This warrants the proposed kernel density estimation of fixation point measurements to identify individual PRLs and BCEAs for these patients (M.D. Crossland et al., 2004).

Importantly, a study comparing the NIDEK MP-1 device and the Rodenstock SLO, and a study comparing the NIDEK MP-1 and the Optos OCT/SLO established inter device reliability of fixation stability measurements (Dunbar et al., 2010; Liu et al., 2015). For the MAIA device, Morales et al. provided a large reference database across a wide range of ages, which indicates a slight decrease of fixation stability with an increase in age (Morales et al., 2016). However, pediatric subjects have also exhibited low fixation stability, possibly due to lower attentiveness (Jones et al., 2016).

Besides the aforementioned summary metrics, new quantitative features have been described to describe fixation, which may be applied to predict visual function (e.g., visual field loss). Montesano et al. proposed to incorporate the temporal relationship between points in addition to the spatial pattern of fixation point measurements (Montesano et al., 2018). Specifically, the sequential Euclidean distance (SED, average of the distances between the fixation points) was shown to be associated with visual field loss in eyes with glaucoma in contrast to the conventional BCEA(Montesano et al., 2018). However, independent validation data has not been reported so far.

### 3.3. Fixation in retinal diseases

While the precise selection of the PRL varies among patients with a central scotoma, some common trends are observable. Commonly, patients select a PRL located superiorly in the retina to the area of the scotoma, which results in a superior visual field defect (Crossland and Rubin, 2014; Fletcher and Schuchard, 1997). This is theoretically advantageous for reading as well as spotting inferior obstacles such as steps (Crossland and Rubin, 2014; Sunness et al., 1996). However, these theoretical advantages often do not translate in improved reading speed (Crossland et al., 2005). Moreover, the specific disease also affects selection of the PRL. Patients with Leber hereditary optic neuropathy (LHON) were shown to self-select frequently unfavorable PRL locations (Altpeter et al., 2013). Patients with a central scotoma secondary to Stargardt disease were shown to more consistently select a PRL superior to the scotoma than patients with GA secondary to AMD (Sunness et al., 1996). Interestingly, patients with Stargardt disease tend to select a PRL not at the margin, but at some distance away from the boundary of RPE-atrophy (Sunness et al., 1996). A possible explanation may be that scotomas boundaries were shown to exceed areas of RPE-atrophy in some patients with Stargardt disease (Sunness, 2008). In eyes with diabetic macular edema, the fixation location was previously shown to be independent of the edema characteristics, but strongly associated with the presence of subfoveal hard exudates (Vujosevic et al., 2008).In terms of fixation stability, macular diseases such as AMD and Stargardt diseases exhibit the worst fixation stability, whereas patients with retinitis pigmentosa are more likely to present with stable fixation, as expected due to relative central preservation until late in the disease (Amore et al., 2013; Crossland and Rubin, 2014; Fujii et al., 2002). Patients with glaucoma and diabetic maculopathy exhibit a wide range of fixation stabilities, dependent on disease severity (Dunbar et al., 2010; Montesano et al., 2018). Overall, fixation stability correlates well with other measurements of visual function such as reading speed and BCVA, which underscores the concurrent validity of fixation stability metrics (Amore et al., 2013; Michael D. Crossland et al., 2004).

The precise determinants for PRL selection in a given patient are only partially understood. It has been shown that fixation stability improves markedly over the first year after losing central vision (Michael D. Crossland et al., 2004). These changes explain 52% of the variability in concurrent improvement of reading speed (Michael D. Crossland et al., 2004). Nevertheless, some data suggests that the natural choice of PRL in patients may not necessarily constitute the optimal PRL (Denniss et al., 2017; Timberlake et al., 1987). Specifically, an early hallmark study by Timberlake et al. using the SLO images of pathology showed that asking patients to use a different PRL may improve reading speed (Timberlake et al., 1987). Similarly, Denniss et al. demonstrated in a large cohort of patients with AMD, that the self-selected PRL tends to be located just foveal to a region of relatively normal sensitivity, suggesting that slightly more eccentric fixation may improve vision (Denniss et al., 2017). In conjunction, such findings suggest that the PRL position may be amenable to fixation training.

### 3.4. Eccentric viewing and fixation stability training

Despite of a large number of publications on eccentric viewing training in general, only few randomized trials are available (Gaffney et al., 2014). To the best of our knowledge, no randomized trial on eccentric viewing training employing a commercially available FCP device has been published to date.

Smaller, single-arm studies suggest that auditory biofeedback training aiming to optimize the PRL position and fixation stability may potentially improve fixation stability as well as visual acuity and reading speed (Morales et al., 2015; Tita-Nistor et al., 2009; Vingolo et al., 2009, 2007). This type of training is possible with the MP-1, MP-3 and MAIA device. Alternatively, visual biofeedback training may be performed with the MP-1 and MP-3 device with a checkboard pattern, which flickers upon fixation with the desired PRL (Vingolo et al., 2013). Morales et al. recently evidenced in a two-arm study that biofeedback training results in superior fixation stability when using an examiner-selected PRL (based on retinal sensitivity) as opposed to fixation stability training for the self-selected PRL (Morales et al., 2020).

Although the PRL may be amenable to training, the question of which location to choose for training is non-trivial. A common concept is to identify a location superior to the scotoma in retinal space (to provide a free inferior visual field for unhindered reading), that exhibits retinal sensitivity better than at the self-selected PRL and in proximity to the fovea (in consideration of spatial resolution) (Tita-Nistor et al., 2009). However, the relationship between sensitivity measured by FCP devices and acuity for non-foveal locations in patients with AMD is not strong, even if eccentricity is taken into account (Denniss et al., 2018). This suggests that FCP-measured sensitivity may not be a good basis for PRL selection. It may be advantageous to select a PRL based on a more complex combination of factors prior to training with an FCP device, though this remains to be tested.

## 4. Previous application in clinical studies and trials

**Please note:** If not stated explicitly otherwise, numerical values in the following paragraphs refer to mesopic testing with the MAIA device and a 4–2 threshold strategy in view of the overall data availability.

### 4.1. Early and intermediate age-related macular degeneration

The disease progression from normal aging to early or intermediate AMD is slow and typically not associated with a loss of best-corrected visual acuity (Lim et al., 2012). Intra-session retest-reliability was reported with 95% COR estimates ranging from ±4.12 dB to ±4.4 dB (Welker et al., 2018; Wu et al., 2013). Mesopic retinal sensitivity measured by FCP deteriorates with worsening disease severity from normal to early and then intermediate AMD, correlates with SD-OCT retinal layer thicknesses, drusen thickness and presence of hyperreflective foci as well as integrity of the ellipsoid zone (Cocce et al., 2018; Echols et al., 2020; Hartmann et al., 2011; Wu et al., 2016a, 2015a, 2014). These findings demonstrate the utility of FCP as a marker of early disease progression. However, macular sensitivity did not exhibit significant association with quality of life assessed by the Impact of vision Impairment questionnaire (IReST) (Pondorfer et al., 2019). Regarding ability to detect change, rather wide-ranging estimates for the rate of change for mesopic sensitivity have been reported, ranging from −0.48 dB/year in the LEAD study (sham group) to up to −3.0 dB/year in smaller longitudinal studies (Hsu et al., 2019; Wu et al., 2019c). A long-term study over 6 year in 16 patients reported a mean reduction of sensitivity by 0.61 dB/year for early and by −1.8 dB/year for intermediate AMD, respectively (Vujosevic et al., 2017a). The recently completed 2 year Duke University natural history study on early AMD (Cocce et al., 2018) and the recently initiated EU-funded MACUSTAR study, the ALSTAR2 study, and the NEI-initiated AMD Ryan Initiative Study (ARIS) aim, among validation of other functional and imaging tests, to further asses the rate of sensitivity change in patients and to identify prognostic markers (Curcio et al., 2020; Finger et al., 2019). In prior publications, comparison of mesopic and dark-adapted thresholds revealed systematically greater degrees of rod-dysfunction compared to cone-dysfunction, especially in presence of reticular pseudodrusen and thinning of the outer nuclear layer (Corvi et al., 2019; Pfau et al., 2018a; Saβmannshausen et al., 2018; Steinberg et al., 2016, 2015). Similar results were observed in the Amish Eye Study using the MP-1 device (Nittala et al., 2019).

### 4.2. Choroidal neovascularization secondary to AMD

Clinical trials investigating choroidal neovascularization (CNV) secondary to AMD have been limited to patients with foveal involvement due to the dependence on BCVA as an outcome measure. FCP provides the opportunity to evaluate patients with extrafoveal and peripapillary lesions and may be useful to identify treatment benefits beyond of the marked effect of anti-VEGF therapy (von der Emde et al., 2019b). Intra-session retest-reliability estimates (COR of ±5.99 dB), as well as data supporting discriminant validity among eyes with non-exudative (quiescent), exudative (active) and formerly exudative (inactive) lesion is available (von der Emde et al., 2019b). Moreover, structure function correlation using an artificial intelligence algorithm (von der Emde et al., 2019a), as well as conventional structure function correlation (Sulzbacher et al., 2012), demonstrated concurrent validity. Ability to detect change in terms of mesopic sensitivity was established in longitudinal studies examining the effect of photodynamic therapy (Schmidt-Erfurth and Michels, 2003), and anti-VEGF therapy (Bolz et al., 2010; Prager et al., 2008; Squirrell et al., 2010). Greater degrees of dark-adapted versus mesopic sensitivity losses were reported for patients with CNV secondary to AMD (von der Emde et al., 2019b).

### 4.3. Geographic atrophy secondary to AMD

BCVA acuity is unsuitable as a functional outcome measure in patient with geographic atrophy (GA) until the end-stage of manifestation of atrophy (Csaky et al., 2019). A frequent feature of GA is initial foveal sparing. Foci of GA typically manifest outside of the fovea representing absolute scotomas (Lindner et al., 2017; Sunness et al., 2008). Specifically, the Age-Related Eye Disease Study 2 (AREDS2) Ancillary SDOCT study demonstrated, that 26.4% of patients develop any GA (central or non-central) over a follow-up of 4 years, while only 15.8% develop central GA (Sleiman et al., 2017). Hence, multiple investigators have evaluated fundus-controlled perimetry as an alternative to BCVA (Hariri et al., 2016; Meleth et al., 2011; Pfau et al., 2020c; Phthalmology, 2016; Pilotto et al., 2013; Schmitz-Valckenberg et al., 2004; Scholl et al., 2004; Sunness et al., 1995; Takahashi et al., 2016). Estimates for the retest-reliability (COR ±6.64 dB) have been reported (Pfau et al., 2020c), as well as structure function analyses demonstrating that mesopic sensitivity is especially reduced in the immediate junctional-zone of 375 μm (Pfau et al., 2020c; Schmitz-Valckenberg et al., 2004), and in association with increased autofluorescence (Pilotto et al., 2013; Schmitz-Valckenberg et al., 2004), loss of the outer nuclear layer and/or photoreceptor inner and outer segments (Hartmann et al., 2011; Landa et al., 2011; Pilotto et al., 2013; Takahashi et al., 2016). Ability to detect change could be established in a longitudinal study with a rate of −1.05 dB/year for the MP-1 device using a 10–2 test pattern (Meleth et al., 2011). Fundus-controlled perimetry served as secondary outcome measure in the large-scale, phase-3 studies for Lampalizumab (Holz et al., 2018) and reported (MP-1, 10–2 pattern) an average decline of the mean sensitivity of 1.27 dB (sham group) in 48 weeks (Heier et al., 2020). So-called patient-tailored perimetry patterns allow for an increased density of test-points in the junctional-zone and thus improve the ability to detect change (cf. 5.2 Test patterns) (Pfau et al., 2020c). Dark-adapted sensitivity losses were shown to be greater in proximity to GA compared to mesopic sensitivity losses (Pfau et al., 2020c). A recent important observation has been that (minimal) residual cone- but not rod-function is detectable in areas with loss of retinal pigment epithelium with persistent overlying outer nuclear layer (Pfau et al., 2019). Of note, the retinal sensitivity was only minimal and no rod function could be detected.

### 4.4. Diabetic retinopathy

While foveal-involving diabetic macular edema may be monitored with BCVA, FCP facilitates the quantification of functional loss due to extrafoveal macular edema or generalized ischemic diabetic maculopathy (Comyn et al., 2014; Querques et al., 2014; Rohrschneider et al., 2000). A plethora of structure function analysis studies have underscored the validity of FCP as an important determinant of various types of pathological processes, including macular edema/retinal thickening (Deák et al., 2010; Vujosevic et al., 2017b, 2006), photoreceptor inner and outer segment integrity (Yohannan et al., 2013), hyperreflective spots (Vujosevic et al., 2016), autofluorescence characteristics (Vujosevic et al., 2011), as well as retinal perfusion based on optical coherence tomography angiography (Alonso-Plasencia et al., 2019; Tsai et al., 2019). In type 1 diabetes, electrophysiological changes can be observed prior to fundoscopic signs. Early abnormalities in retinal sensitivity were not observed, However, the study used the MP-1 device, which is unsuitable to quantify early functional loss (cf. 2.1.8 Dynamic range, ceiling and floor effects) (Sacconi et al., 2019).

### 4.5. Central serous chorioretinopathy

In central serous chorioretinopathy (CSCR), concurrent validity could be established based on structure function correlation (Eandi et al., 2015; Fujita et al., 2012; Gerendas et al., 2018; Springer et al., 2006) as well as correlation with sub-scales of the National Eye Institute 25-Item Visual Function Questionnaire (Gerendas et al., 2018). Interestingly, a small study even provided evidence for predictive validity of FCP in CSCR with regard to the future persistence of subretinal fluid (Roisman et al., 2014). Ability to detect change was demonstrated in the prospective, randomized PLACE trial (van Dijk et al., 2018).

### 4.6. Macular telangiectasia (MacTel) type 2

Macular telangiectasia (MacTel) type 2 is a relatively rare disease that manifests initially in the temporal parafoveal region, but with progression can spread to affect an oval macular region that is wider along the horizontal meridian. A large multicenter study using the MP-1 device could quantitatively demonstrate that functional loss is indeed limited to this oval “MacTel area” (approx. 8° horizontally and 4° vertically) regardless of the disease severity (Vujosevic et al., 2018). Given the characteristic spatial localization of this condition, previously used test patterns have often had a higher sampling density in this central oval region. FCP testing is overall well characterized in terms of reliability, validity and ability to detect change (Charbel Issa et al., 2013, 2007; Schmitz-Valckenberg et al., 2009, 2008). A intra-session retest-reliability of (COR) ±7.2 dB has been reported for the central test-point (Wong et al., 2017). Concurrent validity was established based on the tight structure function correlation with areas of loss of the EZ (Heeren et al., 2017; Kihara et al., 2019; Mukherjee et al., 2017) as well as the association to reading acuity (Tzaridis et al., 2019). Moreover, FCP was shown to be more responsive to change over time than BCVA (Heeren et al., 2015). Mesopic FCP was applied as secondary outcome measure in the Ciliary Neurotrophic Factor (CNTF) implant trial (Chew et al., 2019). Rod dysfunction exceeds cone dysfunction in the parafoveal areas, typically affected by MacTel (Schmitz-Valckenberg et al., 2009, 2008). Further, in patients with macular pigment optical density (MPOD) class 1 patients, dark-adapted cyan sensitivity losses were shown to exceed dark-adapted red sensitivity losses (Heeren et al., 2019).

### 4.7. Stargardt disease

Stargardt disease (*ABCA4*-associated retinal degenerations), one of the most common inherited retinal diseases, exhibits a central-to-peripheral disease progression, commonly in conjunction with foveal and peri-papillary sparing (Cideciyan et al., 2009, 2005; Müller et al., 2018). Accordingly, BCVA is inadequate to capture functional loss over time. The ProgStar studies represent the largest collection of retrospective and prospective assessment of the natural history of Stargardt disease. In addition, a number of other groups have assessed patients with Stargardt using FCP. Concurrent validity could be established in the context of the ProgStar study demonstrating a correlation of mesopic sensitivity with BCVA as well as patient age and disease duration (Schönbach et al., 2017). Ability to detect change was also documented by the ProgStar study (MP-1 device, 10–2 grid) with a rate of change of −0.68 dB per year (Ervin et al., 2019; Etienne M Schönbach et al., 2020). In the ancillary ProgStar study SMART (Scotopic Microperimetric Assessment of Rod Function in Stargardt Disease) using the MP-1-S device, a higher rate of scotopic (−1.42 dB/year) versus mesopic sensitivity (−0.63 dB/year) loss over time compared was observed (Ervin et al., 2019). Recently, it has been shown that analysis of the subset of test-points at the scotoma edge improves the ability to detect change over time (Etienne M. Schönbach et al., 2020b).

As an alternative to rectilinear test-patterns, Cideciyan and coworkers proposed the application of the horizontal foveo-papillary profile as test-pattern. Since this profile encompasses the fovea, as well as peripapillary region, it is typically highly representative of the overall disease severity in most patients with Stargardt disease (Cideciyan et al., 2012). Ability to detect change (as well as retest-variability estimates and concurrent validity) have been later established for the horizontal profile in patients with Stargardt disease as part of a longitudinal study (Pfau et al., 2020b). FCP has served as outcome measure in previous clinical studies for Stargardt disease investigating cellular replacement therapies (Mehat et al., 2018), and constitutes a functional outcome measure in multiple ongoing trials (e.g., NCT03992131).

### 4.8. Retinitis pigmentosa

Retinitis pigmentosa, a heterogeneous group of Mendelian disorders characterized by a progressive peripheral-to-central retinal degeneration, necessitates the quantification of the visual field to evaluate vision loss over time. Concurrent validity has been established based on structure function correlation. Specifically, it was shown that the scotoma boundary detected by FCP matches closely the circular boundary of abnormal autofluorescence (‘Robson-Holder ring’) (Fleckenstein et al., 2009; Robson et al., 2003), as well as the boundary of the photoreceptor inner and outer segments visualized by OCT (Greenstein et al., 2012; Wakabayashi et al., 2010). Moreover, retinal sensitivity was shown to correlate closely with the outer retinal thickness (Funatsu et al., 2019). Intra- and inter-session retest data is available for the common X-linked retinitis pigmentosa subtype (*RPGR* gene) with a reported point-wise COR estimate of ±6 dB (Buckley et al., 2020). Ability to detect change could be established in the context of the PREP-1 Study (rate of −0.4 dB/year, MP-1 device, 10–2 grid) (Iftikhar et al., 2018). Moreover, dedicated analysis of test-points at the edge of scotoma has been proposed in the context of *USH2A* retinopathy to optimize the ability to detect change (Charng et al., 2020a).

Ongoing gene therapy trials for retinitis pigmentosa caused by mutations in RPGR are utilizing FCP as outcome measure (e.g. NCT03116113).(Buckley et al., 2020; Cehajic-Kapetanovic et al., 2020).

### 4.9. Choroideremia

Choroideremia (CHM), a rare, inherited, X-linked recessive retinal disease, presents with night blindness and progressive visual field restriction in late childhood and leads typically to severe vision loss in the fourth decade. The retest-reliability has been evaluated for the MAIA device (COR of ±8.7 dB) (Dimopoulos et al., 2016; Jolly et al., 2017). Concurrent validity of FCP has been well established given the close correspondence of the scotomas boundary and the boundary of the residual functional retina as visualized by fundus autofluorescence (Dimopoulos et al., 2016; Jolly et al., 2017), or OCT imaging (Foote et al., 2019). Further, a weak correlation between mean central sensitivity and reading speed has been reported (Jolly et al., 2019). Ability to detect change has been estimated with cross-sectional data for mesopic testing with a 10–2 grid based on an exponential model (Jolly et al., 2017). The absence of the necessity for pupil dilatation and dark adaptation prior to mesopic testing with the MAIA device has also been studied and confirmed for this patient group (Han et al., 2019, 2017). Further, correlation of FCP sensitivity to rod- and cone-full-field thresholds (FST) in choroideremia supports convergent validity (Dimopoulos et al., 2018a). Recently, evidence of functional cones in conjunction with residual outer nuclear layer outside the boundary of RPE-atrophy has been brought forward (Foote et al., 2019). FCP has also been applied to quantify retinal dysfunction in choroideremia carriers (Edwards et al., 2015). In this cohort, mesopic sensitivity was shown to correlate with the degree of loss of RPE autofluorescence (Edwards et al., 2015). Mesopic FCP served as secondary outcome in multiple gene augmentation therapy trials for CHM (Dimopoulos et al., 2018b; Fischer et al., 2020; MacLaren et al., 2014; Simunovic et al., 2017).

### 4.10. Glaucoma

Glaucoma, a progressive optic neuropathy with characteristic morphological changes of the optic disc, ranks among the most common causes of irreversible vision loss worldwide. Visual field testing constitutes the key functional outcome measure in glaucoma, given that other types of visual function such as BCVA are spared initially (Weinreb et al., 2014). While SAP is the gold standard for visual field testing in glaucoma, SAP may miss early visual field defects (‘pre-perimetric glaucoma’) and detection of disease progression in advanced disease may be impeded due to relatively high test-retest variability. Specific subtypes of glaucoma causing localized defects in the central retina can be effectively assessed with FCP (Ratnarajan et al., 2018; Yusuf et al., 2018). Dense circular peripapillary patterns have been explored for early detection of glaucoma with FCP (cf. 5.2 design of perimetry test-patterns), since these may be advantageous to detect fine nerve fiber bundle defects (Convento et al., 2006; Rohrschneider et al., 1996; Wu et al., 2016b). However, this advantage may be outweighed by the presence of high local gradients of sensitivity (such as those associated with localized defects) that often have a higher degree of measurement variability (Wu et al., 2016b). Regarding detection of diseases progression, the precise source of the high test-retest variability of SAP in glaucoma has been attributed to response characteristics of the retinal ganglion cells and/or incorrect stimulus placement in irregular visual fields (Wall et al., 2010; Wu et al., 2019a; Wyatt et al., 2007). The latter source of test-retest variability may be ameliorated by fundus-tracking (Wu et al., 2016b). In comparison to SAP with the Humphrey Field Analyzer, FCP with the COMPASS device was shown to exhibit better test-retest reliability (Montesano et al., 2019; Rossetti et al., 2015). To our knowledge, data regarding ability to detect disease progression is not yet available.

### 4.11. Other macular diseases

Application of FCP has been applied in multiple other retinal diseases beyond the scope of this article. This includes (among other) disease, retinal vein occlusion (Fujino et al., 2020; Kriechbaum et al., 2009; Sachdev et al., 2019), as well as toxic retinopathies and inflammatory diseases (Fang et al., 2017; Martínez-Costa et al., 2013; Szepessy et al., 2019; Youssef et al., 2017).

## 5. Best practices and clinical trial design

### 5.1. Best practices

The accuracy and reliability of FCP is significantly affected by test procedure and processes, with several factors having an impact on the outcome. Although, FCP is considered an automated procedure, the operator has significant control over the outcome of the test. This section will go through these factors and discuss further with the aim of providing practical advice to optimize data collection.

#### 5.1.1. Adaptation state of the retina and the patient

Early FCP studies employed a variety of adaptation times before conducting the test in order to account for a mixed rod-cone response and allow for adequate rod adaptation. However, several recent studies have shown that standard mesopic FCP is largely a measure of cone function (Crossland et al., 2012; Han et al., 2019; Simunovic et al., 2016) and therefore adaptation before conducting FCP is not required if the patient has only had exposure to ambient lighting. This has been specifically demonstrated in the setting of choroideremia (Han et al., 2019). Patients with rod-cone dystrophies subjectively report higher comfort when tested after some level of dark adaptation, therefore 2–5 min may be offered to aid patient compliance (Han et al., 2019). We also recommend that room lighting is switched off and background luminance is restricted (<1 lux) to ensure that the test luminance parameters are not altered artificially but examiner movement around the room is still facilitated. Later models of the MAIA are outfitted with a red filter to fit over the screen, whose role is to block short-wavelength light from bleaching rod photoreceptors. Similarly, the MP-3 Type S screen may be operated in low-light, red-shifted mode to preserve a scotopic environment.

Generally, FCP testing should be performed prior to imaging. However, if the test is preceded by bright light exposure such as photography or a slit lamp examination, then 10 min of dark adaptation is recommended to allow bleached cones to recover and ensure all patients are at the same level of retinal adaptation before testing (Han et al., 2019).

Scotopic FCP is a relatively new procedure and has limited investigation into best procedures to be followed. As guidance, we recommend dark adaptation of at least 20 min for normal subjects and patients with retinal diseases not affecting the rate of rhodopsin regeneration (Lamb and Pugh, 2004), whereas longer periods of dark adaptation are warranted for diseases that affect Bruch’s membrane interchange such as AMD, or proteins of the visual cycle (Lamb and Pugh, 2004). For AMD patients, 30 or 40 min of dark adaptation have been previously used in FCP studies in consideration the burden for elderly patients (Pfau et al., 2020c; Steinberg et al., 2015). However, longer dark adaptation may be preferable to ensure complete dark adaptation for all patients (Chen et al., 2019; Luu et al., 2018; Owsley et al., 2007).

#### 5.1.2. Pupil dilation

The technical specifications for the MAIA and MP-3 microperimetery devices specify a minimum pupil diameter of 2.5 mm and 4 mm respectively. Under the mesopic conditions most patients do not need to be dilated to meet these requirements (cf. 2.5.2 Maxwellian view versus natural viewing optical systems). Unlike with traditional perimetry, pupil dilation does not seem to degrade FCP performance (Han et al., 2017). However, we do recommend that in a clinical trial setting all subjects should be tested in the same manner, i.e. all participants should be dilated or all un-dilated during all visits.

#### 5.1.3. Set up, instruction, and practice examinations

Lubricating eye drops (artificial tears) should be offered to all patients with an unstable tear film. To ensure a comfortable position for the patient, the perimeter should be on a height adjustable table and set to a comfortable height. Generally, the distance between the chair and forehead rest should be set-up to result in minimal forward pressure against the forehead rest, which tends to be easier to hold than a neutral position. Patient stability and comfort will increase compliance as head movement is less likely.

Careful instruction should be provided on the purpose of the examination and the process involved (Glen et al., 2014). Instruct the participant to look at the fixation target or towards the center of the target if a circle is used. Most machines incorporate an additional large fixation area for patients with poor central visual acuity. However, these will alter the fixation stability recording (Bellmann et al., 2004). Newer models also allow the use of a peripheral fixation target. The author has also used the SLO image to verbally guide fixation in patients with low vision with great success. Although some devices provide auditory feedback when the eyes move out of alignment, the observer should monitor and optimize alignment throughout the examination.

**Fig. 3.**
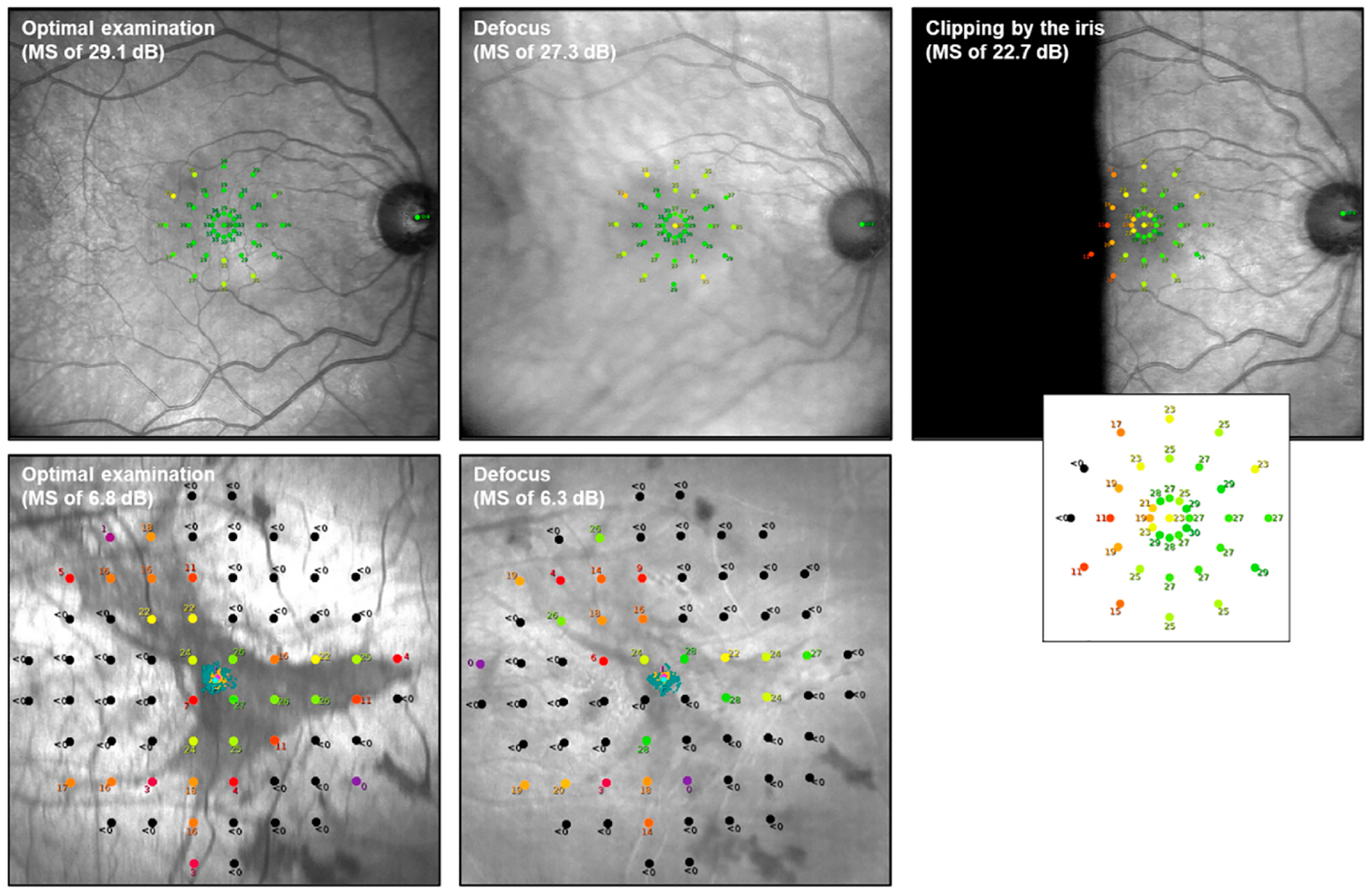
Best-practice recommendations.

During the set-up period, the SLO/fundus image should be centered and fill the screen. The auto-alignment function often requires additional adjustment. The focus can be set by moving the eyepiece forward and backwards. The auto-focus function often requires additional manual input. In other imaging modalities, the importance of optimal focus has been established (Issa et al., 2012). The same applies in FCP as the focus sets the plane in which sensitivity is tested so cannot be changed once started. The accuracy of the focusing also will dictate the accuracy of the sensitivity measurement (Fig. 3). For follow-up examinations the focus should match the initial examination as much as possible to ensure the tests are comparable and reduce variability. In the authors experience, reliability can also be reduced by the patient becoming over stressed or over accommodating. This manifests as an intermittently blurred image with no apparent movement.

Further, the automatic grid placement for the initial examination, as implemented in all devices, will center the grid on the PRL of the first 10 s. Accordingly, the operator should emphasize correct fixation during this time window. For patients with unstable fixation, the manual grid placement options of the device must be used.

Unlike for BCVA testing (Bach and Schäfer, 2016), the effect of auditory feedback originating from the machine has not been studied systematically for FCP. However, previous work on training in perimetry showed that patients valued continuous instruction and feedback (Glen et al., 2014). Moreover, Allison M. McKendrick and coworkers recently demonstrated in the setting of SAP that auditory feedback by a human operator (or a humanoid robot) enhances the subjective experience of perimetry test, while not altering the test results (McKendrick et al., 2019). Accordingly, the patient should be frequently reassured and encouraged with updates provided on test progress. This encourages co-operation and keeps the patient alert.

Perimetry is a difficult test and requires training to perform accurately. In both healthy eyes and disease, practice examinations are required in order to avoid biased results due to a learning effect (Ratnarajan et al., 2018; Wu et al., 2013). Likewise, the European Glaucoma Society guidelines recommend in the setting of SAP a re-test for patients with borderline test results for their first test, as well as three tests per year during the first two years after diagnosis to document progression (European Glaucoma Society, 2017). Thus, we recommend for clinical trials using FCP that the first test to be discarded to increase accuracy of the results.

Fatigue will reduce the reliability of the testing so should be accounted for when setting the study protocol. Visual field testing is an intense test requiring significant patient concentration. In the authors center, we prioritize FCP testing to be conducted early in the test day or following a break in the day to ensure the patient is as refreshed as possible. Breaks can be offered during testing but it can be difficult to realign the patient accurately, particularly if they have low vision and reduced ability to see the fixation target. Fatigue is more likely to occur in the presence of disease and with a longer test time (Johnson et al., 1988; Wildberger and Robert, 1988). Patients with moderate visual field loss associated with depressed sensitivities across the visual field have longer test times which is a factor to consider when planning for fatigue effects.

#### 5.1.4. Test choice

A number of test options are available as standard. Custom tests can easily be created and uploaded into most fundus controlled perimetry devices. The choice of test will be determined by the research or clinical question. The most common test configurations appearing in the literature on assessments of mesopic FCP are the rectilinear 10–2 grid and radial patterns (cf. 5.2 Design of perimetry test-patterns).

For follow up exams, the follow up mode should be used to ensure stimulus presentation the identical location (even for patients with unstable fixation).

#### 5.1.5. Early termination of a test

To minimize patient burden, it is sometimes helpful to terminate a test prematurely and repeat the test. Common reasons are displacement of the test pattern or obvious errors of the threshold determination of the first four test-points. For some devices, the first four thresholds define the initial intensity for the staircase procedure for the remaining test-points in the given quadrant. Accordingly, false-positive responses among the first four test-points may result in excessively long exam durations.

Moreover, an incorrect initial intensity determination for a given quadrant may result in erroneous threshold determinations at neighboring test points, since it would take many more “correct” responses at these locations to converge to the true threshold. For example, if a region is truly scotomatous and the initial presentation starts at 24 dB, six true-negative responses are required to reach a final value of <0 dB. Thus, the likelihood of a false-positive response affecting the results in regions with a deep scotoma are higher compared to regions with normal sensitivity.

#### 5.1.6. Operator training

During testing, there is a lot of information on the operator screen that should be monitored to ensure high quality data is collected. The camera image will provide information on the eye position and movement with an auditory cue when the eye position falls outside of tolerance. The cause of this change in position must be determined. In some cases, it may be head tilt or movement resulting in non-contact with the forehead rest, a change in accommodative effort of the eye, poor positioning at the machine making it difficult to keep the head still, or, true movement in the X, Y or Z axes. Each of these present in a subtly different way and involve differential instructions to the patient to remedy. The individual points being tested are shown in real time and when the response button is pressed and registered, this information is also shown on the screen. Further, false-positive responses to catch-trials at the optic nerve head must be recognized by the operator in order to provide adequate ongoing instruction. Test progression in terms of points with convergence of the staircase (i.e., determined threshold) is reported as a percentage, allowing positive encouragement about progress to be reported to the patient.

This is a lot of information that must be monitored in real time by the operator to ensure test reliability and patient comfort. Previous work on complex screens has shown change blindness to occur with operators missing key information (Durlach, 2004). This establishes the importance of adequate training to conduct FCP. Key strategies to improve performance include attention to the relevant details, being able to discern when a relevant change has occurred such as a blindspot catch-trial, order of features being attended, experience, training on the specific interface, markers alerting problems, and the minimization of distractions and interruptions (Durlach, 2004). Some features are going to vary between patients. For example, some patients will alter their fixation more often so eye movement will require greater attention than other factors. Other patients are more likely to become fatigued or impatient and require frequent updates on progress. Greater experience of conducting FCP allows the operator to learn the factors that require greater attention and hone in on these. Working in a quiet room free of distractions is helpful to both the operator and the patient. There are several alert markers presented, both visual and auditory. However, some of the visual cues such as the color change around the frame of the fundus image can be quite difficult to see through the red filter and may require greater attention when these are variable and require more robust monitoring.

#### 5.1.7. Key points

The accuracy of FCP testing can be improved by following a few key procedural points:

1. Adequate training of the operator and adequate instruction as well as practice for the patient.
2. Proper test set up and image focusing.
3. Ten minutes adaptation to ambient lighting following intense light exposure.
4. Consistency in dilation state between visits in a clinical trial setting.
5. Careful consideration of the testing order.
6. Ensure correct grid placement
7. Careful monitoring of the reliability factors and adequate continuous instruction to correct any errors.

### 5.2. Design of perimetry test-patterns

**Fig. 4.**
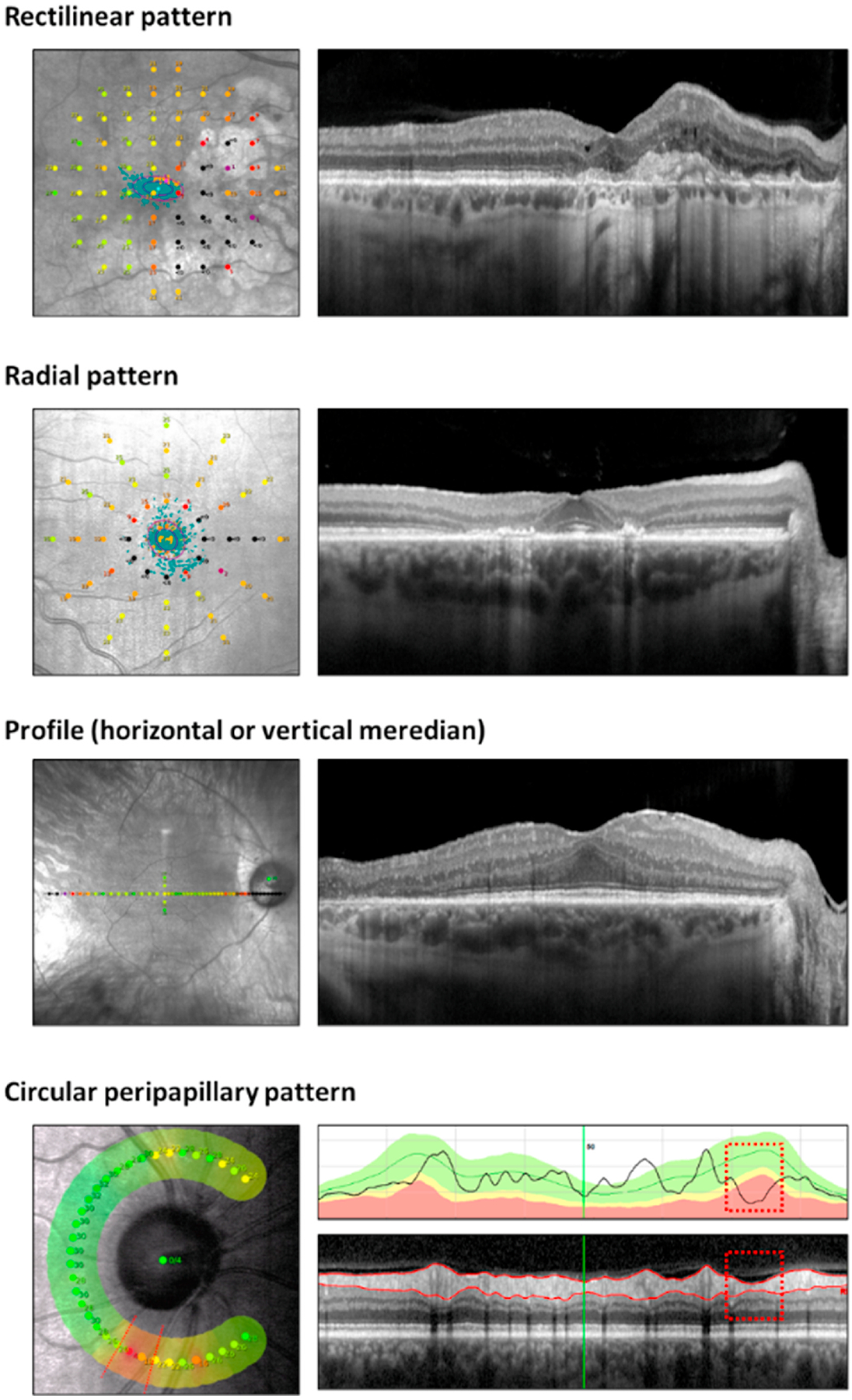
Fixed perimetry patterns.

In principle, two overarching categories of test patterns are conceivable: Fixed and patient-tailored patterns. Hereby, the test-points of fixed patterns are located across all patients at the same position in relation to ocular landmarks (fovea and optic disc). This may be achieved by testing of patients with foveal fixation or through manual positioning of the test pattern. Fixed patterns simplify analysis markedly, since mean sensitivity loss and pattern deviation may be easily calculated with normal data obtained with the same respective pattern (Fig. 4).

Displaced “fixed patterns” (i.e., application of a fixed pattern in a patient with eccentric fixation and without re-positioning of the test pattern) may be considered as a special case of a patient-tailored pattern. For such displaced test-patterns, analysis strategies employing spatially interpolated normative data are also necessary (cf. 5.2.1 Patient-tailored test-patterns).

#### 5.2.1. Fixed test-patterns

Fixed test patterns can be further subdivided in (1.) rectilinear grids, (2.) radial grids and (3.) profiles, which are typically centered to the fovea, and (4.) circular peripapillary patterns:

1. The most commonly applied rectilinear grid in FCP is the 10–2 grid. Due to the uniform spacing of stimuli, indices such as the mean sensitivity or mean deviation are reflective of the underlying visual field and therefore easy to interpret (Pfau et al., 2018b). Generally, the 10–2 grid should constitute the preferred choice in acquired and inherited retinal diseases that affect the entire macula (Fig. 4) (Jolly et al., 2017). However, the test duration using a 10–2 grid is often long with the currently available staircase strategies.
2. In contrast, radial patterns (Fig. 4), which are also commonly applied in FCP, will result in spatially-weighted averages. This weighting increases the influence of foveal and parafoveal regions with higher sampling density in the indices. Accordingly, comparison among results generated with various radial patterns is challenging. It may however be advantageous for the establishment of concurrent validity, since the majority of visual function tests are more strongly associated with central retinal function compared to peripheral function (e.g., BCVA). Of note, topographic modeling may be applied to derive spatially-unweighted visual indices even for data acquired using radial test-patterns (Josan et al., 2020; Weleber et al., 2015).
3. Profiles along the horizontal or vertical meridian (Fig. 4) constitute the third category of fixed patterns. Especially in inherited retinal diseases with a somewhat uniform centrifugal or centripetal progression such as retinitis pigmentosa, disease progression is reflected well by the function along the horizontal meridian. Moreover, relevant regions in Stargardt disease including the degree of foveal and peripapillary sparing may be investigated along the horizontal meridian (Cideciyan et al., 2012).
4. Circular peripapillary patterns (Fig. 4) may be applied in the context of glaucoma and other diseases of the optic nerve head to detect peripapillary nerve fiber bundle defects (Convento et al., 2006; Rohrschneider et al., 1996; Wu et al., 2016b). Dense sampling of the peripapillary region using FCP may allow detection of functional losses missed with the coarse rectilinear patterns for glaucoma (Convento et al., 2006; Rohrschneider et al., 1996), and may be easily correlated to the common circular SD-OCT retinal nerve fiber layer thickness scans (Wu et al., 2016b).

A special case of rectilinear patterns are composite rectilinear patterns as previously applied in Macular Telangiectasia Type 2 (Charbel Issa et al., 2007). A denser test-point spacing for the central macula (2° spacing) compared outer macula (4° spacing) was applied in consideration of the disease topography (Charbel Issa et al., 2007). Similar to radial patterns, the mean sensitivity of such a pattern must be considered as spatially-weighted mean.

A second special case is the use of a rectilinear grid with a very high density in combination with a very coarse staircase strategy or no staircase strategy (i.e., fixed intensity suprathreshold testing). This approach, which provides high spatial resolution, but low resolution in terms of sensitivity, has been proposed to map deep scotomas in a time efficient manner (Wu et al., 2019a).

#### 5.2.2. Patient-tailored test-patterns

Recently, patient-tailored perimetry grids have been introduced in the setting of FCP (MacLaren et al., 2014; Pfau et al., 2020c). In the first gene therapy trial for choroideremia, a rectilinear grid was modified through addition and removal of test-points to cover the island of functional retina, while keeping the overall test-duration short (MacLaren et al., 2014). While this allowed function to be assessed within a given eye over time, this procedure was not automated and did not consider the normative hill-of-vision to compute loss-of-function (rather than absolute sensitivity) for inter-eye comparisons.

**Fig. 5.**
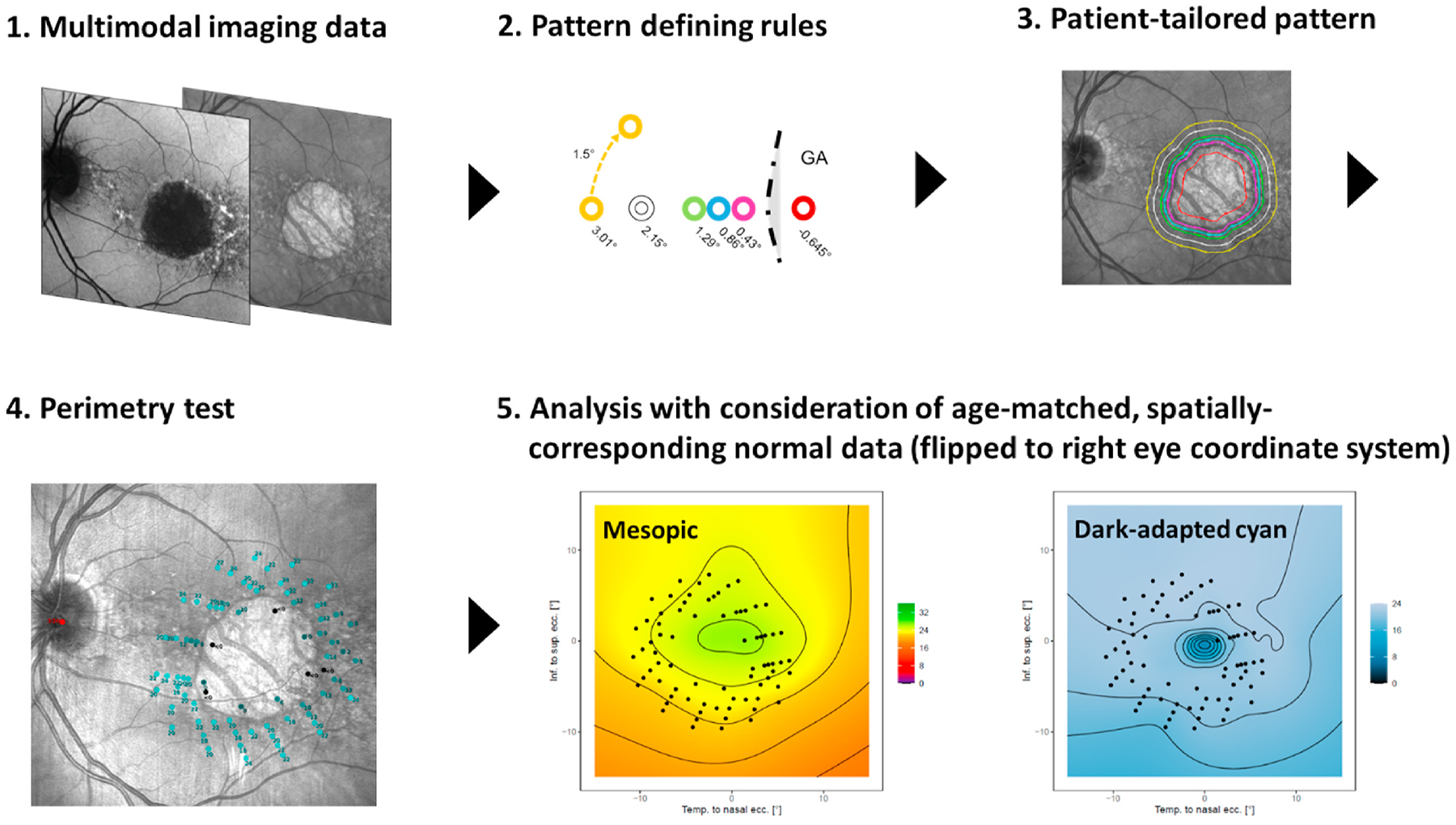
Patient-tailored perimetry.

As a prerequisite for refined patient-tailored perimetry (Fig. 5), a method to spatially-interpolate normative data has been brought forward, which allows to calculate perimetry indices independent of the test pattern and centration (Denniss et al., 2017; Denniss and Astle, 2016). This now allows to analyze data from patients with “decentered fixed patterns” (Denniss et al., 2017; Denniss and Astle, 2016). Building on this, it is now possible to generate and evaluate patient-tailored perimetry patterns based on multimodal imaging data in an automated manner. Hereby, the goal is to maximize the density of test-points in disease relevant regions of interest (e.g., junctional zone in eyes with GA), while minimizing the number of test-point in uninformative regions (e.g., area of atrophy lesion in eyes with GA) (Fig. 5). This fundamental principle of using imaging data to automatically generate patient-tailored patterns could of course be extended to other retinal diseases to measure the progression of well-demarcated boundaries (e. g., late-onset Stargardt disease) (Pfau et al., 2020c). An inherent downside of patient-tailored perimetry is that distant regions are not monitored – however, these may eventually show disease progression as well (i.e., *de novo* foci of atrophy).

### 5.3. Analysis of sensitivity data, statistical considerations for clinical trial planning

The approach for the analysis of FCP sensitivity results in clinical trials can benefit from consideration of how visual field sensitivity data is analyzed more broadly. The first consideration is whether this outcome is analyzed at the individual level (i.e. comparing the difference in how many eyes demonstrate visual function progression based on a specific criteria), or at the population level (i.e. examining whether the overall change in outcome measure over time differs between different intervention groups). In order to be accepted as an outcome measure, the analysis method must be accepted by the regulatory authority and be able to be applied across international multiple sites. The complexity of any analysis methodology must be balanced with the validity of the measurement for what is being measured and ability to pick up relevant changes in the retinal sensitivity.

#### 5.3.1. Analysis at the individual level

For detection of progression at the individual level, there are typically four approaches taken: event-based vs. trend-based analysis, at a global- or point-wise level (Vianna and Chauhan, 2015). The two most common methods used is point-wise event-based analysis (based on a number of tested locations showing deterioration exceeding test-retest limits on several consecutive tests from baseline) or global trend-based analysis (slope of change significantly exceeding zero or age-expected changes).

However, one recent study showed that rigorously-matched comparisons of the point-wise event- and global trend-based analysis actually had similar performance overall in glaucoma eyes, but there was only a moderate level of agreement between the two methods (Wu and Medeiros, 2018a). This suggests that both methods are likely useful at capturing localized and global changes in visual sensitivity, and both should be considered.

A previous study demonstrated that the use of Bayesian models, which allow the results from point-wise event-based analysis to influence the inference of the trend-based analysis (i.e., combining the two approaches), can improve the ability to detect progression that using either method alone (Medeiros et al., 2012). This notion of using both sources of information for detecting progression can also be exploited by using artificial intelligence-based methods. A recent study demonstrated that this method performs better than using either methods alone (Yousefi et al., 2018).

Nonetheless, these novel approaches would require robust development and validation when applied to analysis of FCP sensitivity data in clinical trials before they can gain acceptance. Conventional point-wise event-based or global trend-based analyses would thus currently be preferable, and especially the latter given the current lack of robustly validated methods for pointwise event-based analyses and the similarity of the performance of these two approaches. For such analyses, data should be acquired using the follow-up mode to ensure that the same location is probed across all visits. Indeed, regulatory authorities such as the Food and Drug Administration (FDA) in the United States accepts analyses of visual field endpoints in glaucoma based on either of these two approaches (Weinreb and Kaufman, 2009). Point-wise changes may also be lacking in robustness until the fundus-tracking rate is improved (see discussion in 2.5.1).

#### 5.3.2. Analysis at the population level

While analyses of progression at the individual level in clinical trials is beneficial because it provides a meaningful assessment of treatment efficacy at the person-level, this is achieved at a substantial cost to the power to detect treatment effects.

One recent study demonstrated that an approximately seven to ten fold reduction in sample size requirements could be achieved by evaluating the difference in rate of change in visual function between groups, as compared to evaluating the difference in the number of eyes that showed progression at the individual level based on point-wise event-based analysis, since the latter does not make full use of all the data available over the entire follow-up duration (Wu et al., 2019b). As such, this approach has been used increasingly for analyzing FCP data in clinical trials (Chew et al., 2019; Heier et al., 2020; Wu et al., 2019c).

#### 5.3.3. Other considerations in clinical trials

In addition to the approach for analysis of the FCP sensitivity data, considerations about other aspects of the trial design could improve the power to detect change over time for this outcome measure.

The design of the clinical trial follow-up paradigm will also play an important role in the power to detect differences between group for the outcome measure of FCP sensitivity. Based on previous suggestions that a clustered testing paradigm (i.e. more tests at the bookends of the trial follow-up period) could improve detection of progression at the individual level (Crabb and Garway-Heath, 2012), a modification to the traditional evenly-spaced testing paradigm was made to a landmark clinical trial in glaucoma (Garway-Heath et al., 2015). One recent study showed that this strategy indeed improves the power to detect differences in visual field outcomes in clinical trials, and the power increases the more tests are included at the bookends of the follow-up period (Wu and Medeiros, 2018b).

#### 5.3.4. Strategies used in clinical trials to date

A variety of outcome measures are currently reported for interventional trials. The change in mean sensitivity over time has been applied in the context of the choroideremia gene therapy trials (Xue et al., 2018). For retinal diseases with central RPE-atrophy such as geographic atrophy or Stargardt disease, variegated measures have been proposed including change in mean sensitivity (Meleth et al., 2011; Schönbach et al., 2017), or change in the number or percentage of scotomatous loci (Meleth et al., 2011; Pilotto et al., 2013; Wu et al., 2019a). More complex analyses have also been proposed. This includes classification of test-points as dense scotoma, edge of the scotoma loci and remaining loci based on baseline sensitivity and then computing sensitivity changes for these regions separately (Chen et al., 2011). This selective analysis of test-points at the edge of the scotomas was recently shown to be much more responsive to disease progression for patients with Stargardt disease (Etienne M. Schönbach et al., 2020b) as well as for patients with *USH2A* retinopathy (Charng et al., 2020a).

Moreover, topographic modeling has been recently applied in the setting of SAP and FCP to derive volumetric visual indices as outcome measure for clinical trials (Josan et al., 2020; Parker et al., 2016; Weleber et al., 2015) (NCT00749957, NCT01233609).

#### 5.3.5. Additional strategies developed

In addition to the measures of central tendency (mean sensitivity, mean defect, mean deviation), measures of visual field irregularity (loss variance, pattern standard deviation) are established in the context of SAP. To date, this is not mirrored in commercially-available FCP devices, with the exception of the COMPASS device (Rossetti et al., 2015), and the local defect map provided by the MP-1 device. Typically, sensitivity is shown at each location tested using a color coding that does not take into account patient age or the eccentricity. Both of these are factors that affect what can be considered normal (Cassels et al., 2019; Charng et al., 2020b; Pfau et al., 2018b). Pattern deviation can be calculated for FCP data based on normative data derived through bootstrapping and/or geospatial statistical techniques (Cassels et al., 2019; Denniss and Astle, 2016; Pfau et al., 2018b). In eyes with significant overall sensitivity loss (or total deviation), spatially-resolved mapping of pattern deviation is helpful to delineate scotoma boundaries (Denniss et al., 2017).

Moreover, innovative approaches that take into account the test-retest variability and complex relationships among test-points using compute vision techniques may further increase the sensitivity to localized changes in the visual field (Alexander et al., 2012; Wilson et al., 2018).

### 5.4. FCP and structure function correlation

#### 5.4.1. Overarching aim of structure function correlation

Structure function correlation typically serves two fundamental purposes: (1) establishment of the concurrent validity between functional biomarkers and expert-based classifications (i.e., Clinician-Reported Outcome Assessments [ClinROs]) or native (anatomical) imaging biomarkers, or (2) to document structure function dissociation. The former (establishment structure function correlation) constitutes an important factor for the acceptance of a PerfO by regulatory agencies. Evidence of structure function dissociation can be helpful (2.1) to identify a target for therapeutic intervention, (2.2) as early marker of disease progression and (2.3) as indicator of unanticipated/adverse treatment effects.

Many of the currently used structural biomarkers such as the quantification of RPE-atrophy in eyes with GA are supported by FCP in terms of concurrent validity (Meleth et al., 2011; Schmitz-Valckenberg et al., 2004). Many other biomarkers that are currently quantified in larger trials have also been previously validated using FCP including the central area of residual ellipsoid zone (EZ) presence in RP (Greenstein et al., 2012; Hood et al., 2011), or loss of EZ in MacTel type 2 (Sallo et al., 2012), as well as the integrity of the inner segments and EZ and retinal pigment epithelium-drusen complex (RPEDC) thickness in intermediate AMD (Pfau et al., 2018a; Wu et al., 2014).

Documentation of structure function dissociation serves a three-fold purpose:

1. First, structure function dissociation may allow us to identify diseases that are amenable to vision improvement as recently reviewed in detail by Garafalo et al. in the context of inherited retinal diseases and gene therapy (Garafalo et al., 2020). Briefly, some inherited retinal diseases, including Leber congenital amaurosis (LCA) caused by *RPE65* mutations, exhibited markedly reduced function despite of a relatively preserved retinal architecture. This structure function dissociation becomes apparent when comparing structural data from *RPE6*5-LCA patients with structural data from RP patients. The latter patients exhibit in relative terms markedly better visual function (Cideciyan et al., 2008). By extensions, supervised models, which were trained to predict light sensitivity based on SD-OCT structure in RP patients, may be applied to structural data of *RPE6*5-LCA patients to infer the maximum vision improvement potential through gene therapy in these patients (Sumaroka et al., 2019).
2. Second, structure function dissociation may be exploited to improve diagnostic sensitivity and to obtain a more sensitive measures of diseases progression (Medeiros, 2017; Montesano et al., 2020). Especially in the setting of SAP and glaucoma, multiple studies support the application of composite endpoints of functional and imaging biomarkers to overcome the weaknesses of these endpoints in isolation (Lisboa et al., 2013; Medeiros, 2017; Wu and Medeiros, 2019).
3. Third, structure function dissociation may help to identify unanticipated/adverse treatment effects. Moreover, close structure function association in a natural-history setting may be dissociated in the context of a treatment that preserve tissue anatomy without equally preserving function (Medeiros, 2017). For example, intravitreal injections of 0.1 ml (as used in a recent trial investigating an antibody directed against complement factor D in GA) led frequently to post-injection increases of the intraocular pressure (Holz et al., 2018). While the inner retinal thickness and reflectivity was shown to be of little relevance for the prediction of retinal sensitivity in un-treated patients with GA (Pfau et al., 2020a), this assumption may not necessarily extend to patients with previous post-injection increase of the intraocular pressure. Hence, development of surrogate endpoints requires initially conducting a trial with a given treatment while analyzing the surrogate and true endpoint (Medeiros, 2017).

#### 5.4.2. Technical considerations

For structure function correlation, the image registration and the statistical analysis can markedly affect the results. Importantly, structure function correlation (without image registration) based on stimulus coordinates may lead to erroneous results in presence of obvious eccentric fixation and grid displacement, as well as in patients with subtle eccentric fixation (Mallery et al., 2016).

**Fig. 6.**
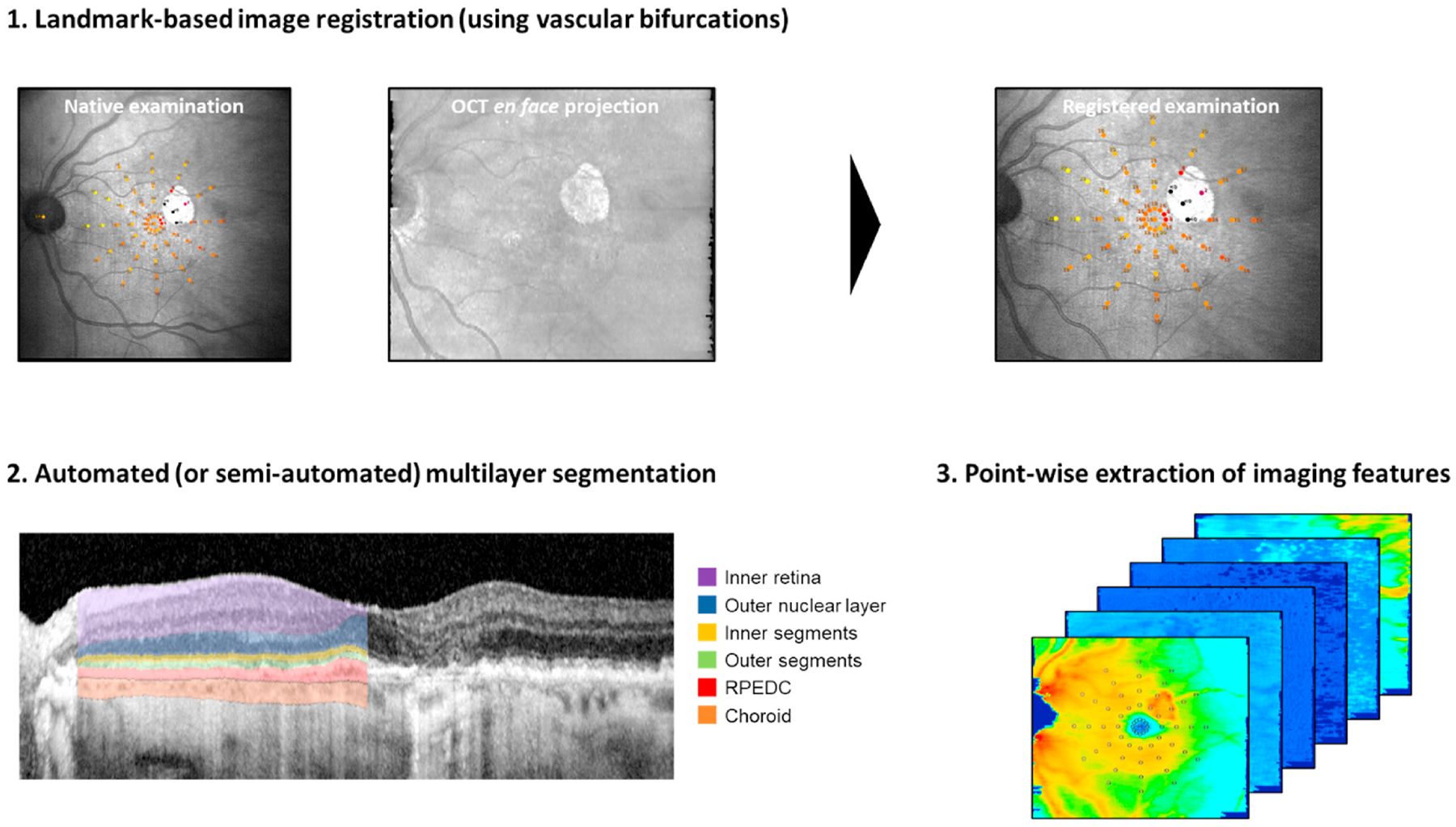
Structure function correlation.

While early reports used rather simple strategies for obtaining structural biomarkers (e.g., for ETDRS subfields (Roh et al., 2019)), it is preferable to conduct structure function analyses in a precise, point-wise manner. This necessitates to register the FCP data to the structural data based on multiple vessel bifurcations (≥4) using ‘nonrigid’ transformations to compensate for differences in field-of-view, head/-eye-rotation in the sagittal, coronal and transverse planes. Subsequently, thickness and reflectivity values may be extracted corresponding to the precise stimulus location (Pfau et al., 2018a). For the Heidelberg Spectralis device it is important to note, that the co-acquired IR image and SD-OCT volume may be minimally offset (Barteselli et al., 2013). Accordingly, the FCP data should be (whenever possible) registered to the actual SD-OCT volume using an *en-face* SD-OCT image rather than to the IR image (Fig. 6). Besides of custom workflows using ImageJ (Institutes of Health, Bethesda, Maryland, USA [described for example in the supplement of (Pfau et al., 2020a)]), NIDEK offers a software solution enabling co-registration of MP-3 data with OCT data (from the NIDEK RS-3000 Advance OCT) to extract layer thickness data for each stimulus (Funatsu et al., 2019).

For disease of the inner retina (glaucoma and other ganglion cell disorders), layer thicknesses and reflectivity values should be extracted for the inner retina with consideration of the displacement of retinal ganglion cell (RGC) from their receptive fields. Multiple models for this displacement, which may be applied across patients for structure function correlation, have been published (Drasdo et al., 2007; Sjöstrand et al., 1999; Watson, 2014). In addition, individualized displacement models taking into account SD-OCT parameters have been previously used for structure function correlation in the setting of SAP and glaucoma (Turpin et al., 2015).

For the inferential statistical analyses (i.e., “calculating P-values”), models applicable to repeated measurements such as linear mixed models should be applied (Wu et al., 2016a). The independent unit of observation are patients. Accordingly, simple linear regression analyses or correlation analyses across individual test-points (as unfortunately commonly reported) will lead to incorrectly low P-values (Lazic, 2010).

Similarly, in the setting of more complex models such as machine-learning and deep-learning models, cross-validated accuracy estimates should be reported for patient-wise cross-validation, since patient-specific information will markedly increase prediction accuracies (Kihara et al., 2019; Pfau et al., 2020a; von der Emde et al., 2019a). Moreover, estimates such as the mean absolute error (MAE) and root-mean-squared error (RMSE) between sensitivity prediction and FCP measurement and Bland-Altman plots should be provided to allow for assessment of the model accuracy. The coefficient-of-determination (*R*^*2*^) tends to be difficult to compare across studies since it is markedly dependent on the underlying dispersion of the data (i.e., study population).

Last, it is often advantageous to standardize structural SD-OCT data from patients as z-scores using test-point-specific normative data with regard to interpretability (Pfau et al., 2020a; von der Emde et al., 2019a). Otherwise, trivial association may mask disease-specific structure function correlations. For example, non-standardized nerve fiber layer thickness, which is essentially an indicator of eccentricity, may carry a paradoxically high feature importance in outer retinal diseases, which disappears, once the structural data is standardized.

## 6. Future directions and conclusions

Tremendous progress has been made since the introduction of the first commercially-available FCP device thirty years ago. However, FCP does not yet constitute a routine clinical examination or outcome measure to date. A variety of challenges and unmet needs provides the opportunity for further research in this area:

1. Psychometric evaluation in a disease- and device-specific manner is lacking for many diseases. While studies reporting concurrent validity in terms of structure function correlation are common, future research will need to address the magnitude of intra-session versus inter-session test-retest reliability, the effect of the test administrator and auditory feedback on test-results, inter-device reliability and content validity (e. g., against full-field threshold testing), discriminant validity (i.e., disease stages) and predictive validity. Regulatory agencies have stated their preference for functional outcome measures that relate to orientation and mobility, daily living in patients’ home environments, and occupational needs such as reading (Csaky et al., 2017; Jolly et al., 2019). This warrants the assessment of concurrent validity beyond structure function correlation. An example for an ongoing natural-history study evaluating specifically these aspects is the EU-funded MACUSTAR study for AMD (Finger et al., 2019), and the NEI-funded ARIS.
2. Multiple developments in the area of standard automated perimetry have not been translated to FCP. These include time-saving threshold estimation strategies such as SITA, ZEST, GATE (COMPASS device provides ZEST), which would facilitate a more widespread adoption of FCP (Turpin et al., 2003). Other examples are the inclusion of pre-adaptation (i.e., dark adaptation) and chromatic perimetry for photoreceptor-specific testing as well as stimulus-presentation techniques to isolate post-receptoral mechanisms. Maximization of photoreceptor and post-receptoral pathway isolation is a prerequisite for hypothesis-based testing of therapeutic efficiency (Simunovic et al., 2016). These approaches are for example needed to differentiate between minimal degrees of cone function from rod intrusion in gene therapy trials for primary cone diseases (e.g., achromatopsia, blue-cone monochromacy) (Thompson et al., 2020).
3. Statistical methods to summarize FCP could be refined and standardized. For example techniques such as spatial-interpolation could be used to obtain unweighted perimetry indices for radial test pattern (Denniss and Astle, 2016; Weleber et al., 2015). Moreover, variability-weighted indices (as used in the context of standard automated perimetry) and point-wise analysis may be more sensitive to functional loss over time (Pfau et al., 2018b; Wu et al., 2016a).
4. The application of parameter-rich models (“artificial intelligence”), including convolution neural networks and random forest regression, has recently allowed multiple groups to drastically increase the accuracy of structure function correlation in the context of macular telangiectasia type 2 (Kihara et al., 2019), Leber congenital amaurosis (Sumaroka et al., 2019), neovascular AMD (von der Emde et al., 2019a) and geographic atrophy (Pfau et al., 2020a). This facilitates *en face* mapping of “inferred sensitivity” with a spatial resolution and retinal coverage beyond the possibilities of psychophysical testing (Pfau et al., 2020a). Extension to further diseases and software solutions for routine clinical practice and clinical trials will be needed.
5. Adaptive-optic (AO) FCP allows for true “microperimetry” with single photoreceptor stimulation (Reiniger et al., 2017). While the utility of dissecting function at a cellular level for basic research is obvious, applicable clinical trial protocols for AO FCP are lacking. To date, it is unclear whether the advantage in resolution of AO FCP truly translates to an improved ability to detect change, which also depends of the dynamic range of stimuli and retinal area investigated. However, recent developments may allow for a much greater dynamic range for AO-based perimetry devices (Domdei et al., 2018).
6. Standardization for testing (test-pattern, staircase strategy, adaptation, pupil dilatation), criteria for test repetition as well as reporting standards are lacking. A well-established example for this type of standardization in the setting of retinal electrophysiology are the guidelines by the International Society for Clinical Electrophysiology of Vision (Robson et al., 2018).

**Fig. 7.**
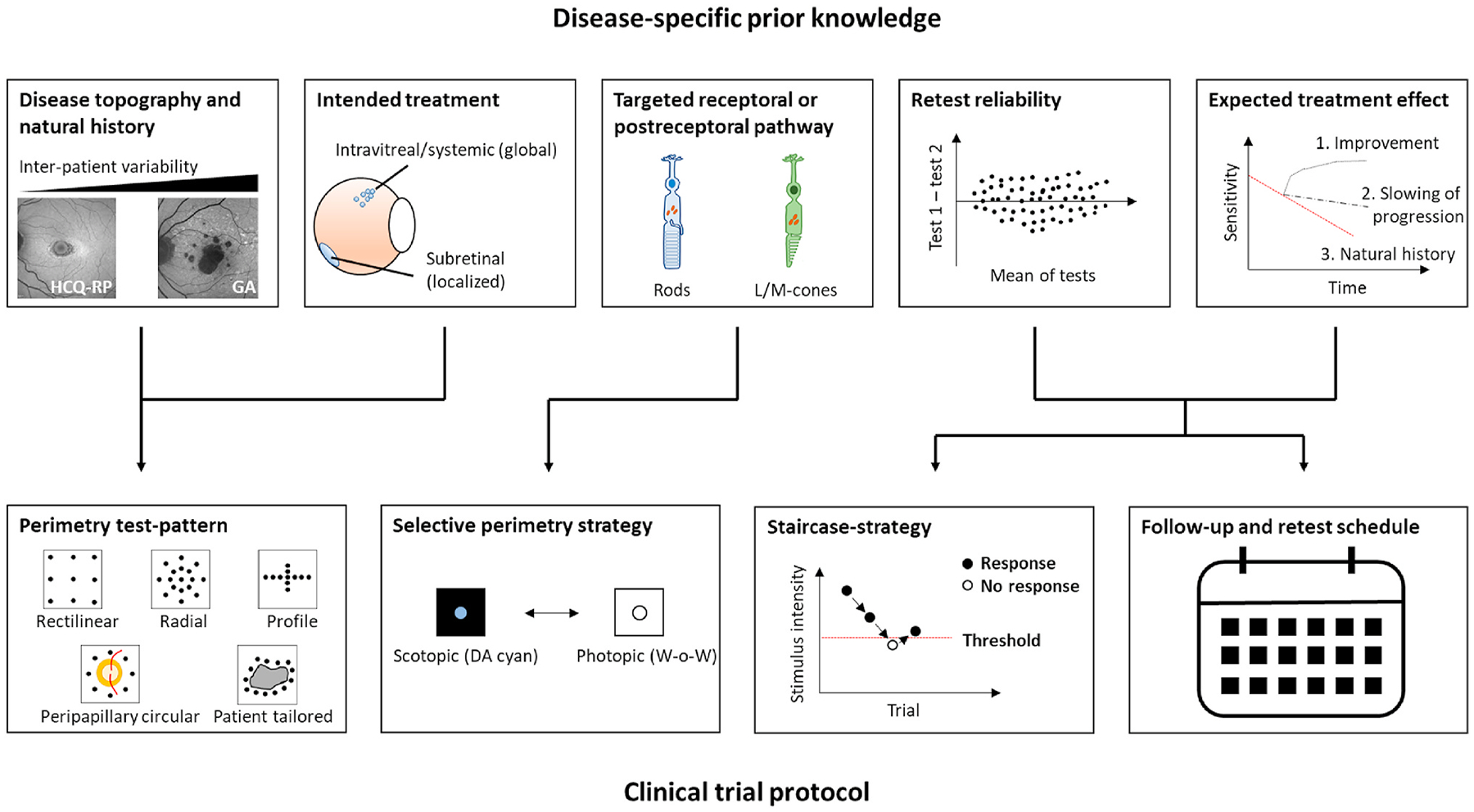
Clinical trial recommendations for fundus-controlled perimetry (FCP).

In summary, we advocate for the application of FCP as an important functional outcome measure for clinical trials. Herein, we provided best-practice recommendations regarding the test-setting and test-pattern. However, prior to the application in clinical trials, natural history studies should underscore the reliability, validity and ability-to-detect-change in a disease-, stage- and device-specific manner. An overview of diseases in which FCP has been (substantially) validated is provided. The selection of the test-pattern and background illumination should be reflective of the topographic progression of the disease and its hypothesized effect on retinal photoreceptors (Fig. 7). Moreover, the statistical analysis strategy and follow-up protocol should be selected in accordance of the expected treatment effect (global vs. local, slowing of disease progression vs. improvement of vision).

**Panel A**. Commercially-available fundus-controlled perimetry (FCP) devices (“microperimeters”) feature three core components. The stimulus and background projection unit employs typically a liquid crystal display (LCD) or a combination of light emitting diodes (LEDs). The imaging unit, which is typically based on an infrared scanning (IR) laser ophthalmoscope (SLO) or IR fundus-camera, simultaneously images the fundus. This imaging is used to track the retina based on landmarks (e.g., vessel bifurcations) in order to adjust the stimulus position or pause the stimulus presentation in moments of fixation instability.

**Panel B**. The background has a marked influence of the measure photoreceptor function. The first and second generation of devices typically project a mesopic background (1.27 cd/m^2^, approximately luminance level of “scene lit by full moon”). The resulting redundancy in stimulus detection may impede detection of minor degrees of isolated rod or cone function loss(Simunovic et al., 2016). More recent devices allow for isolated testing of rod function (scotopic background, <0.03 cd/m^2^, “dark night in the new moon phase”) and cone function (photopic background, 10 cd/m^2^, “daylight”).

Besides mesopic testing, which results in redundancy of target detection (i.e., cone- and rod-mediated) (Simunovic et al., 2016), selective perimetry strategies are available in the setting of fundus-controlled perimetry (FCP) (Pfau et al., 2017b; Remky and Elsner, 2005; Rohrschneider et al., 2008).

**Panel A**. Dark-adapted testing with no background (i.e., scotopic testing) and a short-wavelength stimulus allow for selective probing of rod photoreceptor function with a magnitude of isolation of approximately 2.3 log units (depending on the precise stimulus wavelength).

**Panel B**. Photopic testing with white stimuli (white-on-white testing [W-on-W]) and red stimuli (red-on-white [R-on-W]) is s L/M-cone mediated. Using a yellow chromatic adapting background light and short-wavelength stimulus (blue-on-yellow testing [B-on-Y]), S-cone isolation 1 to 2 log units may be achieved (varies in dependence of the background and stimulus wavelength) (Demirel and Johnson, 2000; Roman et al., 2019). The curves in the lower left panel are based on the dark-adapted spectral sensitivity curves (Wald, 1945), the curves in the lower right depict the population weighted cone sensitivity functions (Stockman and Sharpe, 2000). Dashed arrows highlight the degree of isolation for a given photoreceptor subtype and stimulus wavelength.

The upper row shows a healthy subject and the lower row a patient with choroideremia. Poor focus will result in moderately lower sensitivity measurements (mean sensitivity [MS]) and impedes structure function correlation. Please note the absence of central vascular bifurcations, which could be used for multimodal image registration, in the eye of a patient with choroideremia. Clipping by the iris (i.e., decentration of the device in relation to the eye) can yield also erroneous results. The inlay shows the test results with a white background. For two test-points, no responses were recorded (i.e., sensitivity estimate of <0 dB).

The panel shows typical fixed test patterns employed in clinical studies. The conventional **rectilinear pattern** provides an even sampling density. Accordingly, the mean sensitivity across all test-points constitutes an unbiased estimate of the area examined. The here shown 10–2 grid is applicable across a wide range of retinal disease. However, given the relatively wide gaps between test-points (2°), the 10–2 pattern may be relatively insensitive to slow scotoma progression. Radial patterns are characterized by a central condensation of test-points. Therefore, the mean of all test-points (without correction) will constitute a spatially-weighted average. **Radial patterns** are especially useful to evaluate function in diseases that are confined to the fovea and parafovea including the patient with Chloroquine retinopathy as shown in this example. **Profiles** (i.e., most commonly the horizontal profile) are applicable in diseases that exhibit either a marked centrifugal or centripetal pattern of progression. Especially in retinitis pigmentosa as in this example using a profile test pattern, the sensitivity along the horizontal meridian may be considered as a proxy of overall disease severity. **Circular peripapillary patterns** allow for monitoring of nerve fiber layer bundle defects as commonly observed in glaucomatous optic neuropathy.

In some diseases such as geographic atrophy secondary to age-related macular degeneration, the topographic variability would require a large fixed test-pattern resulting in a burdensome test, while providing only limited information regarding zones of interest with future disease progression (i.e., junctional zone). The number of test-points in these regions essentially determines the ability to detect change. Patient-tailored perimetry allows to maximize the number of relevant test-points while limiting the number of overall test-points. Based on multimodal imaging data, areas of atrophy are annotated (1). An automated software can then use these annotations in conjunction with pre-defined “pattern defining rules” (2) to generate a patient-tailored pattern (3), which is then transferred to the device for perimetry testing (4). To allow for meaningful between-subject comparison of data, all test-points must be analyzed considering age-matched, spatially corresponding normal data (5). To do so, age-adjusted, interpolated sensitivity maps and precise landmark-based (fovea, optic nerve head) registration of data are prerequisites.

For precise structure function correlation, (1) the fundus-controlled perimetry data should be registered to the structural data based on multiple vessel bifurcations (≥4) using ‘nonrigid’ transformations to allow to compensate for differences in field-of-view, head/eye-rotation in the coronal as well as axial plane. In the case of optical coherence tomography (OCT) data, (2) semi-automated or automated multilayer segmentation can be applied. In the last step (3), layer thickness and reflectivity values corresponding precisely to each stimulus area and position can be extracted.

As highlighted throughout this manuscript, a wide range of critical decisions are necessary for the development of clinical trial protocols employing FCP. Disease-specific prior knowledge allows to narrow down these choices. Specifically, the disease topography and natural history in conjunction with the intended treatment effect (localized vs. global) dictate the test-pattern. For example, diseases with a rather consistent spatial manifestation (e.g., retinitis pigmentosa, hydroxychloroquine retinopathy [HCQ-RPE]) may be monitored along the horizontal meridian (profile). In contrast, disease with marked topographic variability (e.g., geographic atrophy [GA]) may be monitored more accurately using a wide-spread rectilinear or patient-tailored pattern. To avoid redundancy in target detection, a selective perimetry strategy should be applied in consideration of the targeted receptoral or postreceptoral pathway. The retest reliability and expected treatment effect (slowing of progression vs. improvement) determine the required granularity of the staircase-strategy and the follow-up as well as intrasession retest schedule.

## Supplementary Material

Supplement

## Figures and Tables

**Table 1:** Commercial fundus-controlled perimetry (microperimetry) devices (photopic testing: background of 10 cd/m^2^; mesopic testing: background of 1.27 cd/m^2^).

Microperimeter	Release date	Manufacturer	Imaging for tracking	Further imaging capabilities	Testing field	Range of stimulus intensity	Eye-tracker frequency	Background illumination	Maximum stimulus intensity	Spectral composition of stimuli/projection system
SLO 101 (and SLO 102)	1990	Rodenstock	SLO – IR (infrared diode laser [780 nm])	Fundus Autofluorescence, fluorescein angiography, Indocyanine green angiography	33 ° × 21 °	0–21 dB	50 Hz	10 cd/m2	71 cd/cm2	Helium–Neon laser beam (632.8 nm) Argon-Laser (488 nm and 514 nm)
NIDEK MP-1	2003	NIDEK Technologies	Fundus camera – IR	Color fundus photography	45°	0–20 dB	25 Hz	1.27 cd/m^2^	128 cd/m^2^	Internal LCD display
•NIDEK •MP-1S	2012	NIDEK Technologies	Fundus camera – IR	Color fundus photography	45°	0–20 dB (extendable through neutral density filters)	25 Hz	0.008 asb	0.808 asb	Internal LCD display with 500 nm shortpass filter
OCT/SLO	2006	OPTOS (previously OPKO Instrumentation)	SLO - IR	OCT	29.7°	0–20 dB	8 Hz	10 cd/m^2^	125 cd/m^2^	Organic light-emitting diode screen
MAIA (Macular Integrity Assessment)	2009	CenterVue	SLO - IR	n/a	36°	0–36 dB	25 Hz	1.27 cd/m^2^	318 cd/m^2^	White LED (with two peaks)
•S-MAIA	2017	CenterVue	SLO - IR	n/a	36°	0–36 dB	25 Hz	n/a	2.54 scot. cd/m^2^	Cyan LED (max. 505 nm) Red LED (627 nm)
NIDEK MP-3	2015	NIDEK Co., Ltd.	Fundus camera – IR	Color fundus photography	45°	0–34 dB	30 Hz	10 cd/m^2^, 1.27 cd/m^2^	3183 cd/m^2^	Internal LCD projector
•NIDEK MP-3 Type S	2019	NIDEK Co., Ltd.	Fundus camera – IR	Color fundus photography	45°	0–24 dB including MP-3 options	30 Hz	0.003 asb	0.30513 asb	Internal LCD projector with a band-pass filter centered at 500 nm (FWHM of 20 nm)
COMPASS	2015	CenterVue	SLO fundus imaging	‘True	60°	0–50 dB	25 Hz	10 cd/m^2^	3183 cd/m^2^	White LED (with two peaks)

## Appendix A. Supplementary data

Supplementary data to this article can be found online at https://doi.org/10.1016/j.preteyeres.2020.100907.
