# Supplementary material for: Fundus-controlled perimetry (microperimetry): Application as outcome measure in clinical trials
Source: Prog Retin Eye Res. Author manuscript; Available in PMC 2026 Feb 4. (PMC12872260; doi:10.1016/j.preteyeres.2020.100907)
Supplement: Supplement [file NIHMS2142187-supplement-Supplement.pdf]

Supplementary Table S1. Comparison of stimulus luminance scales for fundus-controlled perimetry devices. Note: For clarity, this table is limited to the effective (clinically relevant) dynamic range. The Zeiss Humphrey field analyzer (HFA) and the CenterVue COMPASS device feature a physical dynamic range of 50 dB, however, very dim increments of light (34 dB or dimmer in terms of HFA units) are beyond the limits of perception of differential light sensitivity (Wall et al., 2010).

Mesopic testing (background of 4 asb)							Photopic testing (background of 31.4 asb)			
**Device**	**NIDEK MP-1**		**CenterVue MAIA**		**NIDEK MP-3**		**NIDEK MP-3**		**CenterVue COMPASS**	
**Value**	**dB scale**	**Luminance increment ΔL [asb]**	**dB scale**	**Luminance increment ΔL [asb]**	**dB scale**	**Luminance increment ΔL [asb]**	**dB scale**	**Luminance increment ΔL [asb]**	**dB scale**	**Luminance increment ΔL [asb]**
			0	*996*	*0*	*1004.75*	*0*	*10000*	*0*	*10000*
			1	*794.33*	*1*	*798.1*	*1*	*7943.28*	*1*	*7943.28*
			2	*630.96*	*2*	*633.96*	*2*	*6309.57*	*2*	*6309.57*
			3	*501.19*	*3*	*503.57*	*3*	*5011.87*	*3*	*5011.87*
	0	*400*	*4*	*398.11*	*4*	*400*	*4*	*3981.07*	*4*	*3981.07*
	1	*317.73*	*5*	*316.23*	*5*	*317.73*	*5*	*3162.28*	*5*	*3162.28*
	2	*252.38*	*6*	*251.19*	*6*	*252.38*	*6*	*2511.89*	*6*	*2511.89*
	3	*200.47*	*7*	*199.53*	*7*	*200.47*	*7*	*1995.26*	*7*	*1995.26*
	4	*159.24*	*8*	*158.49*	*8*	*159.24*	*8*	*1584.89*	*8*	*1584.89*
	5	*126.49*	*9*	*125.89*	*9*	*126.49*	*9*	*1258.93*	*9*	*1258.93*
	6	*100.48*	*10*	*100*	*10*	*100.48*	*10*	*1000*	*10*	*1000*
	7	*79.81*	*11*	*79.43*	*11*	*79.81*	*11*	*794.33*	*11*	*794.33*
	8	*63.4*	*12*	*63.1*	*12*	*63.4*	*12*	*630.96*	*12*	*630.96*
	9	*50.36*	*13*	*50.12*	*13*	*50.36*	*13*	*501.19*	*13*	*501.19*
	10	*40*	*14*	*39.81*	*14*	*40*	*14*	*398.11*	*14*	*398.11*
	11	*31.77*	*15*	*31.62*	*15*	*31.77*	*15*	*316.23*	*15*	*316.23*
	12	*25.24*	*16*	*25.12*	*16*	*25.24*	*16*	*251.19*	*16*	*251.19*
	13	*20.05*	*17*	*19.95*	*17*	*20.05*	*17*	*199.53*	*17*	*199.53*
	14	*15.92*	*18*	*15.85*	*18*	*15.92*	*18*	*158.49*	*18*	*158.49*
	15	*12.65*	*19*	*12.59*	*19*	*12.65*	*19*	*125.89*	*19*	*125.89*
	16	*10.05*	*20*	*10*	*20*	*10.05*	*20*	*100*	*20*	*100*
	17	*7.98*	*21*	*7.94*	*21*	*7.98*	*21*	*79.43*	*21*	*79.43*
	18	*6.34*	*22*	*6.31*	*22*	*6.34*	*22*	*63.1*	*22*	*63.1*
	19	*5.04*	*23*	*5.01*	*23*	*5.04*	*23*	*50.12*	*23*	*50.12*
	20	*4*	*24*	*3.98*	*24*	*4*	*24*	*39.81*	*24*	*39.81*
			25	*3.16*	*25*	*3.18*	*25*	*31.62*	*25*	*31.62*
			26	*2.51*	*26*	*2.52*	*26*	*25.12*	*26*	*25.12*
			27	*2*	*27*	*2*	*27*	*19.95*	*27*	*19.95*
			28	*1.58*	*28*	*1.59*	*28*	*15.85*	*28*	*15.85*
			29	*1.26*	*29*	*1.26*	*29*	*12.59*	*29*	*12.59*
			30	*1*	*30*	*1*	*30*	*10*	*30*	*10*
			31	*0.79*	*31*	*0.8*	*31*	*7.94*	*31*	*7.94*
			32	*0.63*	*32*	*0.63*	*32*	*6.31*	*32*	*6.31*
			33	*0.5*	*33*	*0.5*	*33*	*5.01*	*33*	*5.01*
			34	*0.4*	*34*	*0.4*	*34*	*3.98*	*34*	*3.98*
			35	*0.32*					*[…]*	*[…]*
			36	*0.25*					*50*	*0.1*
